# Occurrence and diversity of cyanotoxins in Greek lakes

**DOI:** 10.1038/s41598-018-35428-x

**Published:** 2018-12-14

**Authors:** Christophoros Christophoridis, Sevasti-Kiriaki Zervou, Korina Manolidi, Matina Katsiapi, Maria Moustaka-Gouni, Triantafyllos Kaloudis, Theodoros M. Triantis, Anastasia Hiskia

**Affiliations:** 1 Institute of Nanoscience and Nanotechnology, National Center for Scientific Research “Demokritos”, Patr. Grigoriou E’ & Neapoleos 27, 15341 Athens, Greece; 2 School of Biology, Aristotle University of Thessaloniki, 54124 Thessaloniki, Greece; 3 Water Quality Control Department, Athens Water Supply and Sewerage Company - EYDAP SA, Athens, Greece

## Abstract

Toxic cyanobacteria occur in Greek surface water bodies. However, studies on the occurrence of cyanotoxins (CTs) are often limited to mainly microcystins (MCs), with use of screening methods, such as ELISA, that are not conclusive of the chemical structure of the CT variants and can be subject to false positive results. A multi-lake survey in Greece (14 lakes) was conducted in water and biomass, targeted to a wide range of multi-class CTs including MCs, nodularin-R (NOD), cylindrospermopsin (CYN), anatoxin-a (ANA-a) and saxitoxins (STXs), using multi-class/variant LC-MS/MS analytical workflows, achieving sensitive detection, definitive identification and accurate quantitation. A wide variety of CTs (CYN, ANA-a, STX, neoSTX, dmMC-RR, MC-RR, MC-YR, MC-HtyR, dm^3^MC-LR, MC-LR, MC-HilR, MC-WR, MC-LA, MC-LY, MC-LW and MC-LF), were detected, with MCs being the most commonly occurring. In biomass, MC-RR was the most abundant toxin, reaching 754 ng mg^−1^ dw, followed by MC-LR (458 ng mg^−1^ dw). CYN and ANA-a were detected for the first time in the biomass of Greek lakes at low concentrations and STXs in lakes Trichonis, Vistonis and Petron. The abundance and diversity of CTs were also evaluated in relation to recreational health risks, in a case study with a proven history of MCs (Lake Kastoria).

## Introduction

Cyanobacteria are photosynthetic prokaryotic organisms, which can rapidly multiply, forming “blooms” in water^1,2^. They are known to produce various metabolites of diverse and mostly unknown function^3,4^ as well as potent toxins, called cyanotoxins (CTs)^5–8^. CTs are compounds with diverse structures and biosynthetic origin (alkaloid, heterocyclic, peptide, aminoacids, etc)^6,7,9,10^ with various modes of toxicity (e.g. hepatotoxic, dermatotoxic, neurotoxic, cytotoxic)^11,12^. They are therefore, potentially harmful to humans and other organisms^13–16^, posing a significant ecological risk to aquatic habitats and to public health^2,17,18^.

Microcystins (MCs) are the most widespread class of CTs detected in fresh waters^9^. They are cyclic heptapeptides (Fig. S1) containing the unusual β-amino acid ADDA ((2 S,3 S,8 S,9 S)−3-amino-9-methoxy-2,6,8-trimethyl-10-phenyl deca-4,6-dienoic acid) which is responsible for their toxicity due to its conjugated diene, the cyclic structure and the non-esterified Glu residue^19–21^. MCs are known to be hepatotoxic. They mainly mediate their toxicity by uptake into hepatocytes, followed by inhibition of protein phosphatases (PPs), which are able to dephosphorylate serine and threonine residues. Inhibition of PPs results in an increased phosphorylation of proteins in liver cells, significantly affecting metabolic pathways, membrane transport, secretion, etc.^19^. At sub-lethal doses MCs are also known to be potent liver tumor promoters^22^ and produce oxidative DNA damage^23,24^. Up to now, over 248 MC variants have been identified and structurally characterized^25,26^. Nodularins (NODs) are pentapeptides with similar structure with MCs. Ten variants have been identified so far, among which Nodularin-R (NOD) is the most frequently found^25,27,28^. Cylindrospermopsin (CYN) is an alkaloid cyanotoxin (Fig. S1) of rising environmental concern, due to its multiple toxicity endpoints, frequency of occurrence and severity of health impacts^29,30^. CYN is mainly hepatotoxic, but potential effects also include genotoxicity, dermatotoxicity, fetal toxicity and cytotoxicity^29–31^. Unlike other CTs (MCs, NODs) that are mostly intracellular in viable cells, CYN is found mostly as extracellular^32^. Anatoxin-a (ANA-a) is a secondary, bicyclic amine alkaloid (Fig. S1), which is highly water-soluble, presenting increased neurotoxicity^33,34^. The (+) entantiomer, which is toxicologically active, has been associated with a number of animal fatalities, including cattle, dogs, bats, pigeons and flamingos^35–37^. Saxitoxins (STXs), also known as Paralytic Shellfish Poisoning toxins (PSPs), are relatively polar compounds and they are considered to present the highest acute toxicity among CTs^37,38^. Over the last century, STXs present in marine shellfish have been associated with numerous human intoxications resulting in numbness, complete paralysis and even death^37^.

Human exposure to CTs can take place mainly through the oral route, i.e. drinking water, contaminated food or accidentally ingested water through recreational activities (swimming). However, inhalation of particles containing CTs or exposure through dermal contact with contaminated water, are also possible routes of exposure^14,39,40^. The World Health Organization (WHO) and the Oregon Public Health Division (OPHD) have set guideline values for cyanobacteria and CTs present in waters used for recreational purposes^41,42^, in order to address possible issues of human exposure and to evaluate the probability of adverse health effects to humans. WHO has also set a provisional drinking water guideline of 1 μg L^−1^ for MC-LR^43^.

Data on the occurrence of toxic cyanobacteria in surface water bodies are increasingly becoming available and guidelines are gradually implemented worldwide. Nevertheless, the characterization of cyanobacteria species and the determination of their abundance in surface water bodies, are not conclusive of the types and amounts of CTs produced under variable environmental conditions. Therefore, determination of CTs present in the surface water is considered essential for a better estimation of the associated risks. From an analytical endpoint, complex sample matrices (i.e. surface water and cyanobacterial biomass) pose significant challenges, requiring time-consuming and elaborate extraction and clean-up procedures^44^. Furthermore, the number of known CTs is constantly growing, while each group of CTs also comprises various congeners (variants) and isomers. Additionally, available analytical standards, surrogate standards and certified reference materials (CRMs) for the determination of CTs, are limited and expensive. The reliable determination of CTs and their congeners is an extremely intricate but essential task, since dominance of one congener over another could influence the overall toxicity^11,45,46^. For example, toxicological studies on mice have shown that MC-LR and MC-LA present similar acute toxicity, but they are 12 times more toxic than MC-RR. MC-YR is almost as toxic as MC-LR and MC-LA, while (MC-7dmLR) is five times less toxic than its methylated variant^45,47^. The same applies for different STX congeners^9,11,48,49^. Therefore, the determination of a wide range of CTs and their variants, using reliable, sensitive, accurate and validated analytical tools, is extremely important.

A comprehensive review of the past studies that have been carried out to investigate the presence and abundance of CTs in Greek lakes can be found in Table S1. The majority of these studies have employed commercialized fast-screening enzyme – linked immunosorbent assays (ELISA)^50–60^. Although this technique is sensitive, it may present increased uncertainty and several limitations. ELISA methods are based on structural recognition of CTs using antibodies. However, they are not suitable for conclusive detection and identification of CTs, as they are amenable to false-positive results, due to matrix interferences and cross-reactivity with other compounds, and they are not able to distinguish between different CT variants^61–63^. For this reason, they are used for “quantitative screening” in order to detect the presence of MCs or other CTs at a level of interest, whereas confirmation and accurate quantitation is carried out with methods employing liquid chromatography coupled with tandem mass spectrometry (LC-MS/MS). In the case of MC analysis, ELISA antibodies that have been raised using an equal mix of MC-LA, MC-LF, MC-LW, MC-YR and MC-WR, are expected to lead to preference for certain MC-variants^63^. Moreover, ELISA using antibodies for the ADDA moiety, may overestimate MC concentrations, since they can also detect free ADDA, or acyclic biodegradation products of MCs^60–62,64,65^. A few studies have also used protein phosphatase inhibition assay (PPIA) kits^51,55,60^, which is a biochemical assay method that gives an indication for “total MCs” based on a functional response, i.e. the inhibition of protein phosphatases PP1 or PP2. Possible sources of error include various matrix effects and false-positive results due to the presence of other compounds which inhibit protein phosphatases^66–69^. The application of Liquid Chromatography (LC) coupled with UV or Diode Array Detectors (DAD), has also been used^51,54,57,60,70–72^ for the separation and identification of MCs, since it provides increased sensitivity but low identification and confirmatory ability.

The drawbacks of biochemical assays (ELISA and PPIA) and LC coupled with DAD, reveal the necessity for the development and application of highly selective, accurate and sensitive methods, based on LC-MS/MS, providing unequivocal detection and definitive structural identification of the compounds, even at trace levels of concentration^73^. Up to now only a few studies have been carried out using LC-MS/MS^60,69,74–76^ for the determination of CTs in Greek lakes. Nevertheless, only few include validation data to support the accuracy of the analytical methods and ensure the reliability of results. Furthermore, these studies focus on a limited number of Greek lakes and only cover a few CTs, out of a wide diversity of CT groups and variants. Recently, Zervou *et al*. (2017) have developed a multi-toxin analytical method, using a dual cartridge SPE assembly, followed by LC-MS/MS, which provided validated information for a wide range of CTs from different classes, i.e. CYN, ANA, NOD and 12 MCs: [D-Asp^3^]MC-RR, MC-RR, MC-YR, MC-HtyR, [D-Asp^3^]MC-LR, MC-LR, MC-HilR, MC-WR, MC-LA, MC-LY, MC-LW and MC-LF. However, it was only applied for samples originating from two Greek lakes^74^. In order to overcome all the above mentioned limitations, a collection of validated methods can be incorporated in a detailed and thorough step-by-step workflow, which can serve as a tool for analytical laboratories aiming to obtain accurate, reproducible and reliable results, for the determination of several CT groups and variants, as well as for different types of sample (water, biomass etc.).

The objectives of this study, were: (a) the determination for the first time of well-known and also less studied CTs (CYN, ANA-a, STX, neoSTX, desmethyl MC-RR (dmMC-RR), MC-RR, MC-YR, MC-HtyR, desmethyl^3^ MC-LR (dm^3^MC-LR), MC-LR, MC-HilR, MC-WR, MC-LA, MC-LY, MC-LW and MC-LF), in water and cyanobacterial biomass samples from 14 Greek lakes, using highly reliable, sensitive and validated analytical methods, (b) the identification and characterization of cyanobacterial species present in the lakes, (c) the evaluation of possible health risks related to the use of lake water for recreational activities, and (d) the selection of a special case study, with a proven history of cyanobacterial occurrence (Lake Kastoria), so as to assess the presence, abundance and diversity of a wide variety of CTs never studied before, at different time periods. In order to achieve the above, we have developed a simple and detailed workflow, integrating several multi-variant and multi-toxin analytical methods, using a variety of clean-up procedures prior to LC-MS/MS. Using this workflow, several cyanotoxins have been unambiguously identified and quantified for the first time in Greece. Additionally, this workflow provides a monitoring toolkit for laboratories around the world, offering a detailed analytical guide for different types of samples and CTs. The application of this toolkit enables the reliable and accurate determination of a wide diversity of CTs in surface water and biomass, aiming to shape future regulations and guidelines, towards common analytical protocols and standards.

## Results and Discussion

### Performance of the methods

The analytical methods, applied in this study are thoroughly described in section 4. The performance of methods (A) for biomass and (B) for filter analysis was evaluated by analyzing a lyophilized sample free of CTs, spiked with a mixture of the target analytes, at two levels (3 and 30 ng mg^−1^). % Recoveries in biomass (Table S2) were found to be between 60.4–134.5%, except for CYN, neoSTX and STX which produced lower recoveries (30.0–35.4%) and MC-LW and dm^3^MC-LR which provided higher recoveries at the lowest spike level (%Recoveries 154.5 and 150.9, respectively). Method precision (Table S2) was also evaluated and %RSD values ranged from 5.8–26.4% for all toxins, except for dm^3^MC-LR (30.2%). Estimated LODs are shown in Table S3, ranging between 0.1–1.0 ng mg^−1^ dw. % Recoveries of targeted toxins in filter analysis were very close to those obtained by the method for biomass analysis.

In the case of method (C) for filtered water, a dual-cartridge SPE-LC-MS/MS analysis was performed^74^. Method trueness (% recoveries) and precision (%RSD) have already been reported^74^. Values have been confirmed for the present study and %recoveries ranged 62.3–97.9%, except for MC-LW (47%). LOD values ranged from 0.8 to 6.5 ng L^−1^. The LODs of STX and neoSTX in method (D) for the determination of STX and neoSTX in filtered water, were 1.0 and 3.0 μg L^−1^, respectively.

The observed effect of the cyanobacterial extracted matrix, on the analysis of the selected CTs (CYN, ANA-a, MC-RR, MC-LR, neoSTX and STX), is shown in Table S4, ranging 7.8–29.5%, except for CYN with an observed average matrix suppression of 68.0%. As far as specificity is concerned, blank samples were analyzed for every method and no interfering peaks were observed close to the retention times (t_R_) of the analytes. Linearity was assessed with linear regression analysis giving coefficients of determination R^2^ >0.980.

### CTs in cyanobacterial biomass

A wide variety of intracellular CTs (16) was identified in biomass samples collected from Greek lakes, as presented in Table 1, including 12 MCs (dmMC-RR, MC-RR, MC-YR, MC-HtyR, dm^3^MC-LR, MC-LR, MC-HilR, MC-WR, MC-LA, MC-LY, MC-LW, MC-LF), CYN, ANA-a, STX and neoSTX. NOD was not detected in the biomass samples from the lakes. It is the first time that the presence of dmMC-RR, MC-HtyR, dm^3^MC-LR, MC-HilR, MC-WR, MC-LA, MC-LY, MC-LW and MC-LF have been reported in the biomass of lake samples from Greece, using a definitive analytical method, such as LC-MS/MS.

**Table 1:** Cyanotoxin occurrence in biomass samples.

Lake	Identified Cyanotoxins in biomass	Cyanobacteria identified
Kastoria 9/2007	dmMC-RR, MC-RR, MC-YR, MC-LR, STX	***Cylindrospermopsis raciborskii***, ***Microcystis aeruginosa***, *Planktolyngbya limnetica*, *Limnothrix redekei*, *Planktolyngbya circumcreta*, *Cyanodictyon imperfectum*, *Merismopedia* spp., *Aphanizomenon issatschenkoi*, ***Anabaena*** **cf**. ***flos*-*aquae***, ***Microcystis panniformis***
Kastoria 9/2014	dmMC-RR, MC-RR, MC-YR, dm^3^MC-LR, MC-LR, MC-HilR, MC-WR, MC-LA, MC-LY, MC-LW, MC-LF, STX	***Μicrocystis aeruginosa***, ***Anabaena*** **cf**. ***flos*-*aquae***, ***Anabaena*** **cf**. *circinalis*, *Planktolyngbya limnetica*, *Aphanizomenon issatschenkoi*, *Pseudanabaena limnetica*, *Microcystis wesenbergii*, ***Microcystis panniformis***, *Cyanodictyon imperfectum*, *Planktolyngbya circumcreta*
Kastoria 10/2014	dmMC-RR, MC-RR, MC-YR, MC-HtyR, dm^3^MC-LR, MC-LR, MC-HilR,MC-WR, MC-LA, MC-LY, MC-LW, MC-LF	***Μicrocystis aeruginosa***, *Microcystis wesenbergii*, ***Microcystis panniformis***, *Anabaena* spp.
Kastoria 10/2015	dmMC-RR, MC-RR, MC-YR, dm^3^MC-LR, MC-LR, MC-HilR, MC-WR, MC-LA, MC-LY, MC-LW	No data available
Kastoria 9/2016	dmMC-RR, MC-RR, MC-YR, dm^3^MC-LR, MC-LR, MC-HilR, MC-WR, MC-LA, MC-LW	No data available
Pamvotis 9/2014	CYN, MC-RR	***Microcystis aeruginosa***, ***Anabaena flos*-*aquae***, *Anabaena* sp., *Planktothrix agardhii*, *Microcystis wesenbergii*, ***Microcystis panniformis***, *Cuspidothrix issatschenkoi*, *Merismopedia warmingiana*, ***Αphanizomenon flos*-*aquae***
Pamvotis 10/2014	MC-RR, MC-YR, MC-LR	***Microcystis aeruginosa***, ***Microcystis wesenbergii***, ***Anabaena*** **spp**.
Kerkini 6/2008	ANA-a, MC-RR, MC-LR, MC-LW, STX, neoSTX	***Aphanizomenon flos*-*aquae***, ***Microcystis aeruginosa***, *Cylindrospermopsis raciborskii*, *Planktolyngbya limnetica*, *Romeria* cf. *simplex*, *Aphanizomenon issatschenkoi*, *Snowella* sp., ***Anabaena*** **cf**. ***viguieri***, *Aphanothece clathrata*, *Chroococcus limneticus*, ***Anabaenopsis elenkinii***, ***Anabaena flos*-*aquae***, ***Microcystis panniformis***, ***Pseudanabaena limnetica***, ***Anabaena aphanizomenoides***, *Pannus* sp.
Kerkini 10/2014	STX	***Cylindrospermopsis raciborskii***, ***Planktothrix agardhii***, *Pseudanabaena limnetica*, *Romeria simplex*, *Myxobaktron hirudiforme*, ***Anabaenopsis elenkinii***, *Aphanizomenon issatschenkoi*, *Merismopedia warmingiana*, *Snowella* sp.,
Zazari 6/2014	MC-RR, MC-YR	***Microcystis aeruginosa***, *Microcystis wesenbergii*, ***Anabaena spiroides***, *Merismopedia warmingiana*
Mikri Prespa 11/2014	MC-RR, MC-YR, dm^3^MC-LR, MC-LR	***Microcystis aeruginosa***, *Anabaena* cf. *lemmermanii*, *Aphanocapsa* sp.
Vegoritis 9/2008	CYN, MC-RR, MC-LR	***Anabaena viguieri***, ***Aphanizomenon flos*-*aquae***, *Chroococcus* cf. *limneticus*, *Aphanothece* sp., *Lemmermaniella* sp., ***Anabaena flos*-*aquae***, *Aphanizomenon issatschenkoi*, ***Microcystis aeruginosa***
Doirani 9/2008	CYN, ANA-a dmMC-RR, MC-RR, MC-YR, MC-LR	***Cylindrospermopsis raciborskii/Raphidiopsis mediterranea***, ***Microcystis aeruginosa***, *Microcystis wesenbergii*, *Planktolyngbya circumcreta*, *Planktolyngbya limnetica*, ***Aphanizomenon issatschenkoi***, ***Aphanizomenon gracile***, ***Anabaena flos*-*aquae***, ***Anabaena aphanizomenoides***
Chimaditis 9/2008	dmMC-RR, MC-RR, MC-YR, MC-LR, MC-HilR	***Microcystis panniformis***, ***Microcystis aeruginosa***, ***Microcystis wesenbergii***, ***Microcystis flos*-*aquae***, *Planktolyngbya limnetica*, *Romeria simplex*, *Pseudanabaena musicola*, *Planktolyngbya circumcreta*, ***Cylindrospermopsis raciborskii***, *Limnothix* sp., *Merismopedia tenuissima*, ***Anabaena flos*-*aquae***, *Aphanizomenon issatschenkoi*
Petron 9/2008	MC-RR, MC-LR, STX	***Cylindrospermopsis raciborskii***, ***Cyanodictyon imperfectum***, ***Planktolyngbya limnetica***, *Planktolyngbya circumcreta*, *Snowella lacustris*, ***Aphanizomenon gracile***, *Chroococcus* cf. *prescotii*, *Merismopedia tenuissima*, *Anabaenopsis cunningtonii*, *Cyanonephron styloides*, ***Anabaenopsis elenkinii***, *Chroococcus limneticus*, *Merismopedia warmingiana*, *Cyanodictyon planctonicum*, *Aphanocapsa delicatissima*, *Radiocystis geminata*, ***Planktolyngbya microspira***, *Merismopedia minima*, *Microcystis wesenbergii*, ***Planktothrix agardhii***
Trichonis (shallow)11/2008	dmMC-RR, MC-YR, dm^3^MC-LR, STX, neoSTX	***Aphanizomenon flos*-*aquae***, ***Planktothrix rubescens***, *Aphanocapsa* cf. *incerta*, *Snowella* sp., *Chroococcus limneticus*
Trichonis (deep)11/2008	dmMC-RR, MC-YR, dm^3^MC-LR, STX	***Planktothrix rubescens***
Volvi 7/2009	MC-RR	*Planktolyngbya limnetica*, *Planktolyngbya circumcreta*, *Limnothrix* cf. *redekei*, ***Cylindrospermopsis raciborskii***, ***Anabaena aphanizomenoides***, ***Anabaenopsis elenkinii***, *Anabaenopsis cunningtonii*, *Snowella* sp., *Aphanizomenon issatschenkoi*, *Jaaginema* sp.
Ismarida 9/2010	—	***Planktothrix agardhii***, *Pseudanabaena* cf. *limnetica*, ***Anabaena*** **cf**. ***viguieri***, ***Anabaena aphanizomenoides***, *Romeria* cf. *simplex*, ***Anabaenopsis elenkinii***, *Merismopedia* sp., *Raphidiopsis mediterranea*, *Aphanizomenon issatschenkoi*
Ismarida 8/2011	—	*Anabaenopsis arnoldii*
Marathonas 9/2013	—	No data available
Vistonis 7/2014	STX, neoSTX	***Aphanizomenon favaloroi***
Vistonis 8/2014	STX, neoSTX	***Aphanizomenon favaloroi***, *Pseudanabaena limnetica*, *Limnothrix* sp.

In bold letters: dominant cyanobacterial species (>10% of total cyanobacterial biomass content).

**Figure 1:**
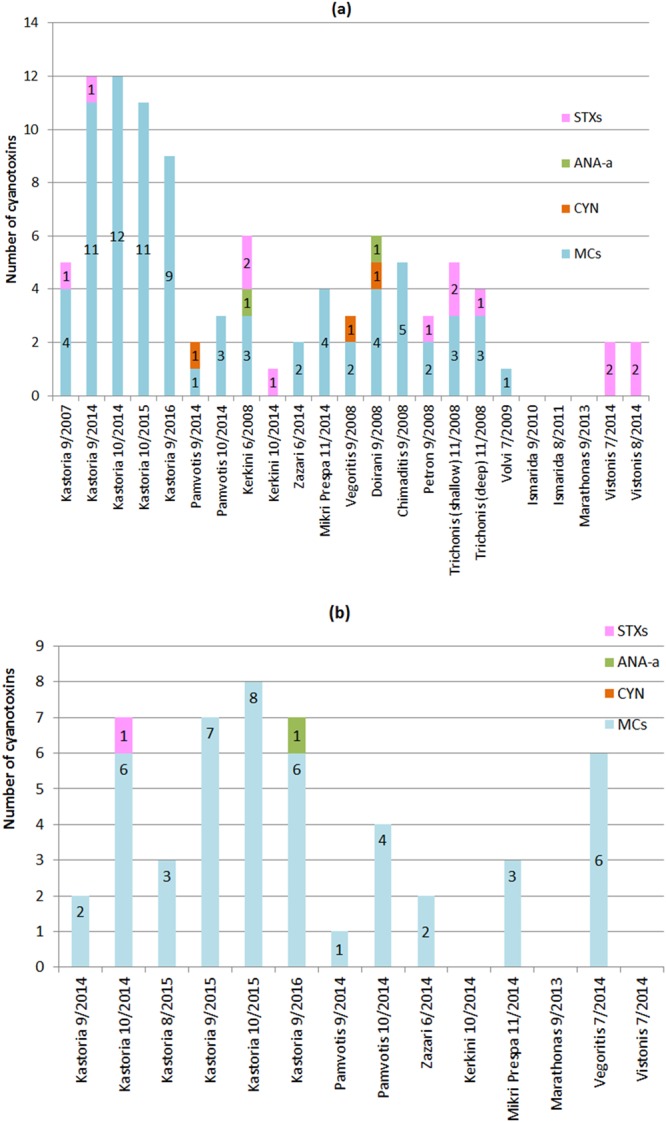
Cyanotoxin occurrence in (**a**) biomass samples, (**b**) water samples, obtained from Greek lakes (number of variants identified for each CT group).

Figure 1a depicts the profile of different groups of CTs i.e. MCs, CYN, ANA-a, STXs identified in the biomass of the studied Greek lakes. Only Lakes Ismarida and Marathonas were free of CTs in the sampled biomass. All the other lakes contained MCs, except for Lake Vistonis (2014) and Lake Kerkini (10/2014). STX was also identified in the biomass of 5 lakes (Kastoria, Kerkini, Trichonis, Petron and Vistonis) and neoSTX was found in 3 biomass samples (Lakes Kerkini, Trichonis and Vistonis). This is the first report of such a wide diversity of CT groups and variants detected in Greek lakes.

Lake Kastoria, presented the highest variety of CTs in its biomass (Table 1) throughout the study. In September 2014, 11 MC-variants and STX were detected in the biomass collected by the lake, which is the most diverse cocktail of toxin variants identified in Greece during the study. Lake Doirani (9/2008), also presented a large diversity of toxins in the biomass, including CYN, ANA-a and 4 MCs (dmMC-RR, MC-RR, MC-YR, MC-LR). In Lake Chimaditis during the same period (September 2008), 5 MCs were identified (dmMC-RR, MC-RR, MC-YR, MC-LR, MC-HilR), while Lake Trichonis presented biomass samples with 3 MCs (dmMC-RR, MC-YR, dm^3^MC-LR), STX and neoSTX. In Lake Kerkini (6/2008), 3 MCs (MC-RR, MC-LR, MC-LW), ANA-a, STX and neo-STX. In all other lakes a smaller number of toxins were identified.

The most commonly found CT group in the biomass of Greek lakes was MCs (found in 11 out of 14 lakes). MC-RR was the most frequently detected MC-variant (10 lakes), followed by MC-LR, MC-YR and dmMC-RR, which were found in 8, 7 and 4 lakes, respectively.

It should be noted that most of the studies conducted in the past have focused on the analysis of CTs in lake samples containing both biomass and water. In most cases, a certain amount of the sample was filtered and the biomass collected was further extracted for CT analysis. The amount of CTs determined in the filtered biomass, was expressed as μg of intracellular toxins per volume (L) of filtered water. During a cyanobacterial bloom, the surface of water can be abundant with biomass (bloom/scum), however, as cyanobacteria are not homogenously distributed in the water volume, this practice can result in over-estimation of CTs per volume of water. Additionally, this approach gives no information regarding the extracellular (water-dissolved) amount of CTs. In the study presented here, we have selected to report separately the extracellular (free water soluble) toxins found in filtered water (μg L^−1^) and the intracellular (cell-bound) toxins found in lyophilized biomass. The content of intracellular toxins is also expressed in two ways, i.e. nanogram of toxins found per milligram of dry biomass (ng mg^−1^ dw) and microgram of toxins found in dry biomass collected by a certain volume of water that was filtered (μg L^−1^). In this way, results can be comparable to other studies. Furthermore, both fractions of the toxin content (free and cell-bound) can be clearly discriminated.

**Figure 2:**
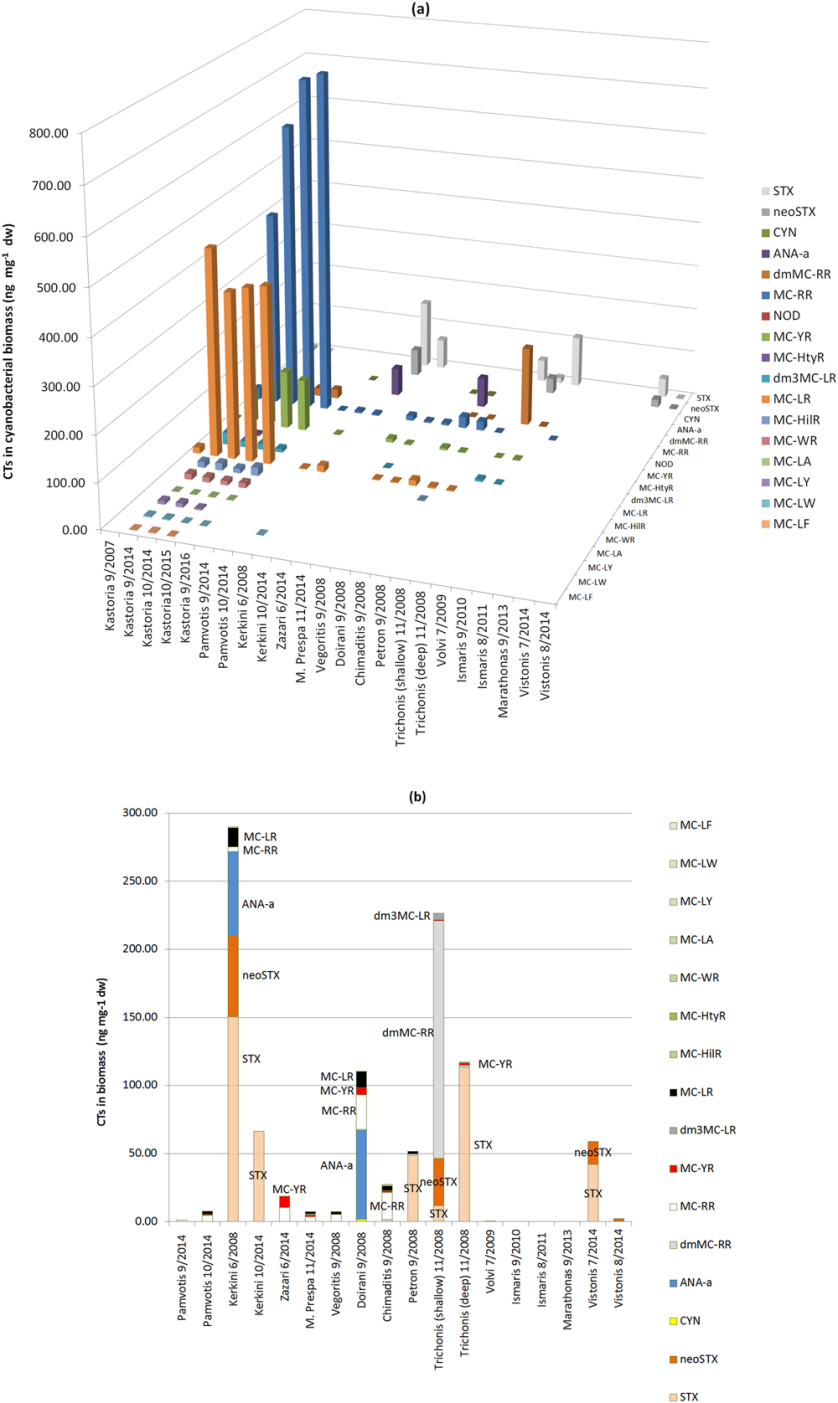
Cyanotoxin occurrence and content in cyanobacteria obtained by (**a**) all Greek lakes, (**b**) all Greek lakes excluding Lake Kastoria.

Table 2 presents the content of intracellular CTs and their variants, found in Greek lakes (ranges in Figure S2). It is evident that biomass samples from Greek lakes are rich in MCs. The results are also graphically presented in Fig. 2a,b. Intracellular MC-RR was found to be the most abundant toxin, reaching 754 ng mg^−1^ dw biomass or 75.4 μg L^−1^ (Lake Kastoria, 9/2016). The mean content of MC-RR in biomass samples from Greek lakes was 177 ng mg^−1^ dw (17.3 μg L^−1^) and the median was 10.5 ng mg^−1^ dw (0.403 μg L^−1^). The second most abundant CT present in cyanobacterial biomass of Greek lakes is MC-LR, found at intracellular content up to 458 ng mg^−1^ dw biomass (45.8 μg L^−1^), in Lake Kastoria (9/2014). The mean content of MC-LR in biomass samples was 138 ng mg^−1^ (13.4 μg L^−1^) and the median reached 12.7 ng mg^−1^ dw (0.205 μg L^−1^). The total MC (TMCs) content of biomass (sum of 12 MCs determined in this study) from Greek lakes ranged from <LOQ to 1316 ng mg^−1^ dw or <LOQ to 132 μg L^−1^ (Kastoria 9/2016). Nevertheless, some of the samples (i.e. Kastoria) presented extremely high content, which increased the overall mean content of the samples. Other MCs detected in the biomass of Greek lakes (dmMC-RR, MC-YR, MC-HtyR, dm^3^MC-LR, MC-HilR, MC-WR, MC-LA, MC-LY, MC-LW, MC-LF) were found in lower levels, as shown in Table 2.

**Table 2:** Content of intracellular cyanotoxins in Greek lakes.

Content of intracellular CTs in Biomass (ng mg^−1^ dw)																	
Sample	STX	neoSTX	CYN	ANA-a	dmMC-RR	MC-RR	NOD	MC-YR	MC-HtyR	dm^3^MC-LR	MC-LR	MC-HilR	MC-WR	MC-LA	MC-LY	MC-LW	MC-LF
Kastoria 9/2007	6.10	n.d.	n.d.	n.d.	<LOQ	24.6	n.d.	2.77	n.d.	n.d.	13.4	n.d.	n.d.	n.d.	n.d.	n.d.	n.d.
Kastoria 9/2014	1.40	n.d.	n.d.	n.d.	34.0	430	n.d.	71.5	n.d.	26.8	458	13.3	12.7	<LOQ	7.46	2.94	<LOQ
Kastoria 10/2014	n.d.	n.d.	n.d.	n.d.	20.9	632	n.d.	93.2	2.94	13.5	368	15.7	11.2	<LOQ	8.73	2.77	2.50
Kastoria 10/2015	n.d.	n.d.	n.d.	n.d.	16.6	738	n.d.	128	n.d.	13.4	382	10.2	9.30	1.32	3.41	<LOQ	<LOQ
Kastoria 9/2016	n.d.	n.d.	n.d.	n.d.	19.9	754	n.d.	113	n.d.	8.00	391	18.7	10.7	<LOQ	n.d.	<LOQ	n.d.
Pamvotis 9/2014	n.d.	n.d.	<LOQ	n.d.	n.d.	1.06	n.d.	n.d.	n.d.	n.d.	n.d.	n.d.	n.d.	n.d.	n.d.	n.d.	n.d.
Pamvotis 10/2014	n.d.	n.d.	n.d.	n.d.	n.d.	4.50	n.d.	<LOQ	n.d.	n.d.	2.12	n.d.	n.d.	n.d.	n.d.	n.d.	n.d.
Kerkini 6/2008	150	59.7	n.d.	61.7	n.d.	3.42	n.d.	n.d.	n.d.	n.d.	14.0	n.d.	n.d.	n.d.	n.d.	<LOQ	n.d.
Kerkini 10/2014	66.5	n.d.	n.d.	n.d.	n.d.	n.d.	n.d.	n.d.	n.d.	n.d.	n.d.	n.d.	n.d.	n.d.	n.d.	n.d.	n.d.
Zazari 6/2014	n.d.	n.d.	n.d.	n.d.	n.d.	10.5	n.d.	8.19	n.d.	n.d.	n.d.	n.d.	n.d.	n.d.	n.d.	n.d.	n.d.
Mikri Prespa 11/2014	n.d.	n.d.	n.d.	n.d.	n.d.	3.54	n.d.	<LOQ	n.d.	<LOQ	<LOQ	n.d.	n.d.	n.d.	n.d.	n.d.	n.d.
Vegoritis 9/2008	n.d.	n.d.	<LOQ	n.d.	n.d.	5.09	n.d.	n.d.	n.d.	n.d.	2.14	n.d.	n.d.	n.d.	n.d.	n.d.	n.d.
Doirani 9/2008	n.d.	n.d.	1.72	65.5	<LOQ	25.6	n.d.	4.95	n.d.	n.d.	12.1	n.d.	n.d.	n.d.	n.d.	n.d.	n.d.
Chimaditis 9/2008	n.d.	n.d.	n.d.	n.d.	1.33	20.1	n.d.	<LOQ	n.d.	n.d.	3.44	<LOQ	n.d.	n.d.	n.d.	n.d.	n.d.
Petron 9/2008	48.7	n.d.	n.d.	n.d.	n.d.	0.84	n.d.	n.d.	n.d.	n.d.	1.96	n.d.	n.d.	n.d.	n.d.	n.d.	n.d.
Trichonis 11/2008 shallow	11.8	34.5	n.d.	n.d.	175	n.d.	n.d.	<LOQ	n.d.	4.93	n.d.	n.d.	n.d.	n.d.	n.d.	n.d.	n.d.
Trichonis 11/2008 deep	113	n.d.	n.d.	n.d.	1.43	n.d.	n.d.	<LOQ	n.d.	<LOQ	n.d.	n.d.	n.d.	n.d.	n.d.	n.d.	n.d.
Volvi 7/2009	n.d.	n.d.	n.d.	n.d.	n.d.	<LOQ	n.d.	n.d.	n.d.	n.d.	n.d.	n.d.	n.d.	n.d.	n.d.	n.d.	n.d.
Ismaris 9/2010	n.d.	n.d.	n.d.	n.d.	n.d.	n.d.	n.d.	n.d.	n.d.	n.d.	n.d.	n.d.	n.d.	n.d.	n.d.	n.d.	n.d.
Ismaris 8/2011	n.d.	n.d.	n.d.	n.d.	n.d.	n.d.	n.d.	n.d.	n.d.	n.d.	n.d.	n.d.	n.d.	n.d.	n.d.	n.d.	n.d.
Marathonas 9/2013	n.d.	n.d.	n.d.	n.d.	n.d.	n.d.	n.d.	n.d.	n.d.	n.d.	n.d.	n.d.	n.d.	n.d.	n.d.	n.d.	n.d.
Vistonis 7/2014	42.0	17.0	n.d.	n.d.	n.d.	n.d.	n.d.	n.d.	n.d.	n.d.	n.d.	n.d.	n.d.	n.d.	n.d.	n.d.	n.d.
Vistonis 8/2014	0.40	1.63	n.d.	n.d.	n.d.	n.d.	n.d.	n.d.	n.d.	n.d.	n.d.	n.d.	n.d.	n.d.	n.d.	n.d.	n.d.
**Mean**	**49**.**0**	**28**.**2**	<**LOQ**	**63**.**6**	**30**.**0**	**177**	**n**.**d**.	**35**.**7**	**2**.**94**	**9**.**68**	**138**	**11**.**8**	**11**.**0**	<**LOQ**	**6**.**53**	<**LOQ**	<**LOQ**
**Median**	**42**.**0**	**25**.**8**	<**LOQ**	**63**.**6**	**16**.**6**	**10**.**5**	**n**.**d**.	**3**.**86**	**2**.**94**	**8**.**00**	**12**.**7**	**13**.**3**	**11**.**0**	<**LOQ**	**7**.**46**	<**LOQ**	<**LOQ**
**Concentration of intracellular CTs in Biomass (expressed in μg L** ^**−1**^ **)**																	
**Sample**	**STX**	**neoSTX**	**CYN**	**ANA-a**	**dmMC-RR**	**MC-RR**	**NOD**	**MC-YR**	**MC-HtyR**	**dm** ^**3**^ **MC-LR**	**MC-LR**	**MC-HilR**	**MC-WR**	**MC-LA**	**MC-LY**	**MC-LW**	**MC-LF**
Kastoria 9/2007	0.100	n.d.	n.d.	n.d.	<LOQ	0.403	n.d.	0.045	n.d.	n.d.	0.219	n.d.	n.d.	n.d.	n.d.	n.d.	n.d.
Kastoria 9/2014	0.140	n.d.	n.d.	n.d.	3.40	43.0	n.d.	7.15	n.d.	2.68	45.8	1.33	1.27	<LOQ	0.746	0.294	<LOQ
Kastoria 10/2014	n.d.	n.d.	n.d.	n.d.	2.09	63.2	n.d.	9.32	0.294	1.35	36.8	1.57	1.12	<LOQ	0.873	0.277	0.250
Kastoria 10/2015	n.d.	n.d.	n.d.	n.d.	1.66	73.8	n.d.	12.8	n.d.	1.34	38.2	1.02	0.930	0.130	0.341	<LOQ	<LOQ
Kastoria 9/2016	n.d.	n.d.	n.d.	n.d.	1.99	75.4	n.d.	11.3	n.d.	0.800	39.1	1.87	1.07	<LOQ	n.d.	<LOQ	n.d.
Pamvotis 9/2014	n.d.	n.d.	<LOQ	n.d.	n.d.	0.106	n.d.	n.d.	n.d.	n.d.	n.d.	n.d.	n.d.	n.d.	n.d.	n.d.	n.d.
Pamvotis 10/2014	n.d.	n.d.	n.d.	n.d.	n.d.	0.450	n.d.	<LOQ	n.d.	n.d.	0.212	n.d.	n.d.	n.d.	n.d.	n.d.	n.d.
Kerkini 6/2008	1.48	0.587	n.d.	0.607	n.d.	0.034	n.d.	n.d.	n.d.	n.d.	0.137	n.d.	n.d.	n.d.	n.d.	<LOQ	n.d.
Kerkini 10/2014	6.65	n.d.	n.d.	n.d.	n.d.	n.d.	n.d.	n.d.	n.d.	n.d.	n.d.	n.d.	n.d.	n.d.	n.d.	n.d.	n.d.
Zazari 6/2014	n.d.	n.d.	n.d.	n.d.	n.d.	1.05	n.d.	0.819	n.d.	n.d.	n.d.	n.d.	n.d.	n.d.	n.d.	n.d.	n.d.
Mikri Prespa 11/2014	n.d.	n.d.	n.d.	n.d.	n.d.	0.354	n.d.	<LOQ	n.d.	<LOQ	<LOQ	n.d.	n.d.	n.d.	n.d.	n.d.	n.d.
Vegoritis 9/2008	n.d.	n.d.	<LOQ	n.d.	n.d.	0.118	n.d.	n.d.	n.d.	n.d.	0.049	n.d.	n.d.	n.d.	n.d.	n.d.	n.d.
Doirani 9/2008	n.d.	n.d.	0.024	0.926	<LOQ	0.363	n.d.	0.070	n.d.	n.d.	0.171	n.d.	n.d.	n.d.	n.d.	n.d.	n.d.
Chimaditis 9/2008	n.d.	n.d.	n.d.	n.d.	0.076	1.16	n.d.	<LOQ	n.d.	n.d.	0.198	<LOQ	n.d.	n.d.	n.d.	n.d.	n.d.
Petron 9/2008	0.159	n.d.	n.d.	n.d.	n.d.	0.003	n.d.	n.d.	n.d.	n.d.	0.006	n.d.	n.d.	n.d.	n.d.	n.d.	n.d.
Trichonis 11/2008 shallow	0.177	0.518	n.d.	n.d.	2.62	n.d.	n.d.	<LOQ	n.d.	0.074	n.d.	n.d.	n.d.	n.d.	n.d.	n.d.	n.d.
Trichonis 11/2008 deep	1.59	n.d.	n.d.	n.d.	0.020	n.d.	n.d.	<LOQ	n.d.	<LOQ	n.d.	n.d.	n.d.	n.d.	n.d.	n.d.	n.d.
Volvi 7/2009	n.d.	n.d.	n.d.	n.d.	n.d.	<LOQ	n.d.	n.d.	n.d.	n.d.	n.d.	n.d.	n.d.	n.d.	n.d.	n.d.	n.d.
Ismaris 9/2010	n.d.	n.d.	n.d.	n.d.	n.d.	n.d.	n.d.	n.d.	n.d.	n.d.	n.d.	n.d.	n.d.	n.d.	n.d.	n.d.	n.d.
Ismaris 8/2011	n.d.	n.d.	n.d.	n.d.	n.d.	n.d.	n.d.	n.d.	n.d.	n.d.	n.d.	n.d.	n.d.	n.d.	n.d.	n.d.	n.d.
Marathonas 9/2013	n.d.	n.d.	n.d.	n.d.	n.d.	n.d.	n.d.	n.d.	n.d.	n.d.	n.d.	n.d.	n.d.	n.d.	n.d.	n.d.	n.d.
Vistonis 7/2014	4.20	1.70	n.d.	n.d.	n.d.	n.d.	n.d.	n.d.	n.d.	n.d.	n.d.	n.d.	n.d.	n.d.	n.d.	n.d.	n.d.
Vistonis 8/2014	0.040	0.160	n.d.	n.d.	n.d.	n.d.	n.d.	n.d.	n.d.	n.d.	n.d.	n.d.	n.d.	n.d.	n.d.	n.d.	n.d.
**Mean**	**1**.**62**	**0**.**741**	**0**.**012**	**0**.**767**	**1**.**32**	**17**.**3**	**n**.**d**.	**3**.**49**	**0**.**294**	**0**.**901**	**13**.**4**	**1**.**17**	**1**.**10**	**0**.**067**	**0**.**653**	**0**.**145**	**0**.**154**
**median**	**0**.**18**	**0**.**552**	**0**.**007**	**0**.**767**	**1**.**66**	**0**.**403**	**n**.**d**.	**0**.**116**	**0**.**294**	**0**.**800**	**0**.**205**	**1**.**33**	**1**.**10**	**0**.**046**	**0**.**746**	**0**.**080**	**0**.**133**

n.d. not detected.

Occurrence of MCs and more specifically the presence of MC-RR, MC-LR and MC-YR in increased abundance in Greek lakes, is in agreement with numerous studies worldwide, showing that the main MC-congeners produced by *Microcystis* spp. are MC-LR, MC-RR and MC–YR in varying proportions^2,10,20^, while more hydrophobic MCs (e.g. MC-LA, MC-LW, MC-LF) and some desmethyl variants are rarely dominant^45,77,78^. Most of the lakes studied were found to be dominated by *Microcystis* sp., *Aphanizomenon* sp., *Anabaena* sp. and *Anabaenopsis* sp. which are known MC producers.

Previous studies have also demonstrated the presence of MCs in Greek lakes. In a study carried out by Gkelis *et al*.^55^ cyanobacterial biomass from several Greek lakes was extracted and analyzed, using ELISA, PPIA and LC-DAD^55^. Results were in agreement with the present study, showing that MCs were found in 95% of the samples, with MC-RR and MC-LR representing the predominant MC variants. In a previous study of six Greek lakes and reservoirs^59^, MCs were also the predominant CT group MC intracellular (cell-bound), where concentration reached a maximum of approximately 40, 15, 8, 70 and 98 μg L^−1^, for Lakes Doirani, Kerkini, Volvi, Kastoria and Pamvotis, respectively. Similar results were also obtained for the same lakes during an older survey (spring 2005) using ELISA for the determination of TMCs^53^. In our study, Lakes Doirani, Kerkini, Volvi, Kastoria and Pamvotis presented intracellular TMCs content reaching 43.1, 18.1, <LOQ, 1316 and 7.51 ng mg^−1^ dw, respectively, which can be also expressed as 0.610, 0.178, <LOQ, 132 and 0.751 μg L^−1^, respectively. For the first time, 12 different MCs were chromatographically separated and identified in these lakes, providing individual MC variant content, using a validated and reliable analytical method (LC-MS/MS). It is evident, that the concentrations obtained by LC-MS/MS determination of different MCs, are generally significantly lower than the ones provided by the previous studies of the same lakes and determined using ELISA methods.

Lakes Kastoria, Mikri Prespa, Pamvotis and Kerkini have also been studied in the past using HPLC-DAD and ELISA^70^, where Lake Kastoria presented a maximum TMCs content of 2564.3 ng mg^−1^, with samples containing MC-LR, [D-Asp^3^]MC-LR, MC-RR, [Dha^7^]MC-RR, MC-YR and MC-LA. Lakes Mikri Prespa and Pamvotis also contained high content of MCs (more than 1100 ng mg^−1^). Lake Kerkini presented MC-LR, MC-RR, MC-YR) at maximum total content of 598.6 ng mg^−1^ and MC-LR in Lake Vistonis at 317.2 ng mg^−1^. In the present study, we are reporting an even wider variety of intracellular MCs in those lakes. Specifically, samples obtained from Lake Kastoria included large amounts of intracellular MCs, while LC-MS/MS analysis revealed, for the first time, the presence of an even larger MC diversity (dmMC-RR, MC-YR, MC-HtyR, dm^3^MC-LR, MC-HilR, MC-WR, MC-LA, MC-LY, MC-LW, MC-LF). Biomass collected for the present study by Lakes Kastoria (2016), Mikri Prespa (2014), Pamvotis (2014) and Kerkini (2008), presented TMC content 1316, 7.19, 7.51, and 18.1 ng mg^−1^ dw, respectively. Finally, no MCs were detected in the present study in samples collected from Lake Vistonis.

The biomass samples that were obtained from Lake Trichonis in November 2008, contained only the desmethyl variants dmMC-RR (reaching 175 ng mg^−1^) and dm^3^MC-LR (reaching 4.93 ng mg^−1^), but not their most commonly-found methylated forms. As shown in Table 1, the lake was dominated by *Planktothrix rubescens* and *Aphanizomenon flos*-*aquae* in the shallow layer while the maximum abundance of *P*.*rubescens* was observed in deeper water^79^. To the best of our knowledge, this is the first report of a Greek lake presenting the maximum abundance of *P*. *rubescens* in deep waters. The presence of desmethyl variants is in agreement with past studies suggesting that *Planktothrix* and some *Anabaena* sp. tend to produce mainly desmethyl MC variants, namely [D-Asp^3^] MC-RR, [D-Asp^3^, Dhb7] MC-RR, [D-Asp^3^] MC-LR and [D-Asp^3^] MC-HtyR^80–82^. Nevertheless, the presence of the desmethyl MC variants could show periodic trends in a lake, alternating between periods in which they represent the only variants and periods when they coexist with other variants of MCs. These variations have been attributed to changes in chemotypes’ composition of *Planktothrix rubescens* in relation to certain environmental variables^82^. The absence of the methylated variants could be attributed to the total absence of *Microcystis* sp., *Cylindrospermopsis raciborskii* or *Anabaena flos*-*aquae*, which are usually responsible for the production of methylated MC variants (Table 1).

The present study for Lake Pamvotis, showed very low intracellular MCs content, reaching a maximum of 7.51 ng mg^−1^ dw during October 2014 or 0.751 μg L^−1^. Furthermore, CYN was detected for the first time in this lake at trace concentration. In the past^83^, cell bound MCs were found in the same lake to reach a maximum of 19 μg MC eq. L^−1^, while free extracellular MCs were detected with the same method, reaching a maximum of 9 μg MC eq. L^−1^. Another study for the same lake using ELISA for the detection of MCs has shown that extracellular MCs ranged from 0.012–7.88 μg MC-LR eq. L^−1^, while intracellular MCs ranged 0.15–15.21 μg MC-LR eq. L^−1^ ^54^. Similar results were also obtained in an earlier study of the Lake Pamvotis^58^, where the measured concentration using ELISA ranged from 0.01 to 19.5 μg L^−1^.

Marathonas, is a drinking water reservoir of Athens, which seasonally suffers from cyanobacterial blooms^84^. In the present study, filtered samples did not contain traceable amount of cyanotoxins, in contrast to past results from our research group^69^, showing that biomass samples obtained during an intense bloom (10/2010) contained MC-RR, MC-YR and MC-LR at levels of 1956, 555 and 382 ng mg^−1^ dw, respectively.

Apart from MCs, other CTs such as CYN, ANA-a, STX and neoSTX were identified in Greek lakes in the present study (Tables 1, 2 and Fig. 2b). CYN was identified in the biomass of lakes Pamvotis (9/2014), Vegoritis (9/2008) and Doirani (9/2008) (Table 1) at trace concentrations. CYN was detected in the past in lakes Volvi, Pamvotis and Karla^59^, using ELISA, without definitive identification using LC-MS/MS. This is the first report of CYN identification in the lakes of Greece, using LC-MS/MS.

ANA-a was detected in lakes Kerkini (6/2008) and Doirani (9/2008), at levels of 61.7 ng mg^−1^ dw and 65.5 ng mg^−1^ dw, respectively. In the past ANA-a has been reported in lake Kerkini (2008) using ELISA^59^. These are the first reports of ANA-a presence in specific Greek lakes using LC-MS/MS.

STX and neo-STX were identified for the first time in biomass samples from Lakes Trichonis, Vistonis and Petron at content values reaching 113.4, 42.0 and 48.7 ng mg^−1^ dw, respectively. This is the first report of STXs occurrence in those lakes. These concentrations can be expressed also as 1.59, 4.20 and 0.159 μg L^−1^, respectively. In Lake Kerkini, STX was detected at 150 ng mg^−1^ dw or 1.48 μg L^−1^. Previous studies employing ELISA methods detected total STXs in Lakes Doirani, and Kerkini, at concentrations ranging from 0.4 to 1.2 μg L^−1^ and also in Lake Pamvotis (1.3–2.1 μg L^−1^)^83^.

### CTs in lake waters

The analysis of filtered water obtained from Greek lakes revealed the presence of various extracellular CTs, mainly MCs (dmMC-RR, MC-RR, MC-YR, dm^3^MC-LR, MC-LR, MC-HilR, MC-LY, MC-WR, MC-LA) as well as ANA-a and STX. NOD was not detected in the filtered water samples from the lakes. Table 3 shows the various CTs found to be present in water. Figure 1b depicts the diversity of different groups of CTs identified in the studied lakes. Most of the analyzed filtered water samples contained mainly MCs.

**Table 3:** Cyanotoxin occurrence in filtered water samples.

Lake	Sampling Date	Identified Cyanotoxins in water
Kastoria	9/2014	MC-RR, MC-LR
Kastoria	10/2014	dmMC-RR, MC-RR, MC-YR, dm^3^MC-LR, MC-LR, MC-HilR, STX
Kastoria	8/2015	MC-RR, MC-YR, MC-LR,
Kastoria	9/2015	dmMC-RR, MC-RR, MC-YR, dm^3^MC-LR, MC-LR, MC-HilR, MC-LY
Kastoria	10/2015	dmMC-RR, MC-RR, MC-YR, dm^3^MC-LR, MC-LR, MC-HilR, MC-WR, MC-LA
Kastoria	9/2016	ANA-a, dmMC-RR, MC-RR, MC-YR, dm^3^MC-LR, MC-LR, MC-HilR
Pamvotis	9/2014	MC-RR
Pamvotis	10/2014	dmMC-RR, MC-RR, MC-YR, MC-LR
Zazari	6/2014	MC-RR, MC-LR
Kerkini	10/2014	—
Mikri Prespa	11/2014	MC-RR, MC-YR, MC-LR
Marathonas	9/2013	—
Vegoritis	7/2014	dmMC-RR, MC-RR, MC-YR, dm^3^MC-LR, MC-LR, MC-LY
Vistonis	7/2014	—

Lake Kastoria presented the highest number and diversity of CTs in nearly all the water samples obtained throughout different time periods (Fig. 1b). The highest variety of MCs in Lake Kastoria, was found during October 2015, when 8 different MCs were detected (dmMC-RR, MC-RR, MC-YR, dm^3^MC-LR, MC-LR, MC-HilR, MC-WR, MC-LA). In the water samples from Lake Kastoria obtained during 2014 and 2016, a large variety of MCs was also found. Table 3 shows the individual MC variants present in each water sample. These results are in close agreement with our previous studies, where several MC variants, e.g. [D-Asp^3^]MC-RR, MC-RR, MC-YR, [D-Asp^3^]MC-LR, MC-LR, MC-HilR, MC-WR, MC-LA and MC-LY were found to be present for the first time in the filtered water of this lake^74^. Water originating from Lake Vegoritis (2014) contained 6 MCs (dmMC-RR, MC-RR, MC-YR, dm^3^MC-LR, MC-LR, MC-LY) and Lake Pamvotis (October 2014) contained 4 MCs (dmMC-RR, MC-RR, MC-YR, MC-LR). In Lake Mikri Prespa (November 2014) MC-RR, MC-YR and MC-LR were detected. Lake Kerkini (October 2014), Marathonas reservoir (9/2013) and Lake Vistonis (9/2014) did not contain detectable amounts of the targeted CTs.

MC-RR was detected in five lakes and was the most abundant toxin in the filtered water samples. The concentration of the water samples are given in Table 4 (ranges in Fig. S3) reaching 338 μg L^−1^ (Lake Kastoria, October 2014) with a mean concentration of 62.1 μg L^−1^ and a median value of 36.5 μg L^−1^. Equally abundant in the filtered water samples was MC-LR, found at concentrations up to 354 μg L^−1^ (Lake Kastoria, October 2014) with a mean value of 69.3 μg L^−1^ and a median of 21.7 μg L^−1^. MC-YR followed, as it reached the level of 80.7 μg L^−1^, with mean and median values of 22.0 and 12.9 μg L^−1^, respectively. In the past, extracellular TMC concentration in Lake Kastoria has been reported to reach 4 μg L^−1^ ^53^ using ELISA. In another study of the same lake during 2015, MC concentrations were equal to 1.7, 63 and 3.6 μg L^−1^, for [D-Asp^3^]MC-RR, MC-RR and MC-YR, respectively^74^, while older investigations detected the presence of MC-RR and MC-LR at much lower concentrations (0.007 and 0.008 μg L^−1^, respectively)^60^.

**Table 4:** Concentration of extracellular cyanotoxins in Greek lakes.

Concentration of extracellular CTs in water (μg L^−1^)																	
Sample	STX	neoSTX	CYN	ANA-a	dmMC-RR	MC-RR	NOD	MC-YR	MC-HtyR	dm^3^MC-LR	MC-LR	MC-HilR	MC-WR	MC-LA	MC-LY	MC-LW	MC-LF
Kastoria 9/2014	n.d.	n.d.	n.d.	n.d.	n.d.	12.3	n.d.	n.d.	n.d.	n.d.	14.8	n.d.	n.d.	n.d.	n.d.	n.d.	n.d.
Kastoria 10/2014	52.4	n.d.	n.d.	n.d.	28.20	338	n.d.	80.7	n.d.	9.60	354	16.1	n.d.	n.d.	n.d.	n.d.	n.d.
Kastoria 8/2015	n.d.	n.d.	n.d.	n.d.	n.d.	0.092	n.d.	0.013	n.d.	n.d.	0.072	n.d.	n.d.	n.d.	n.d.	n.d.	n.d.
Kastoria 9/2015	n.d.	n.d.	n.d.	n.d.	0.007	0.308	n.d.	0.074	n.d.	0.026	0.373	0.019	n.d.	n.d.	0.018	n.d.	n.d.
Kastoria 10/2015	n.d.	n.d.	n.d.	n.d.	1.71	62.9	n.d.	3.60	n.d.	0.034	18.3	0.101	0.507	0.537	n.d.	n.d.	n.d.
Kastoria 9/2016	n.d.	n.d.	n.d.	0.058	0.99	36.5	n.d.	4.65	n.d.	0.076	13.2	1.07	n.d.	n.d.	n.d.	n.d.	n.d.
Pamvotis 9/2014	n.d.	n.d.	n.d.	n.d.	n.d.	2.8	n.d.	n.d.	n.d.	n.d.	n.d.	n.d.	n.d.	n.d.	n.d.	n.d.	n.d.
Pamvotis 10/2014	n.d.	n.d.	n.d.	n.d.	<LOQ	66.9	n.d.	21.1	n.d.	n.d.	131	n.d.	n.d.	n.d.	n.d.	n.d.	n.d.
Zazari 6/2014	n.d.	n.d.	n.d.	n.d.	n.d.	17.5	n.d.	n.d.	n.d.	n.d.	25.0	n.d.	n.d.	n.d.	n.d.	n.d.	n.d.
Kerkini 10/2014	n.d.	n.d.	n.d.	n.d.	n.d.	n.d.	n.d.	n.d.	n.d.	n.d.	n.d.	n.d.	n.d.	n.d.	n.d.	n.d.	n.d.
Mikri Prespa 11/2014	n.d.	n.d.	n.d.	n.d.	n.d.	41.4	n.d.	36.2	n.d.	n.d.	40.8	n.d.	n.d.	n.d.	n.d.	n.d.	n.d.
Marathonas 9/2013	n.d.	n.d.	n.d.	n.d.	n.d.	n.d.	n.d.	n.d.	n.d.	n.d.	n.d.	n.d.	n.d.	n.d.	n.d.	n.d.	n.d.
Vegoritis 7/2014	n.d.	n.d.	n.d.	n.d.	<LOQ	104	n.d.	29.3	n.d.	10.9	96.3	n.d.	n.d.	n.d.	18.1	n.d.	n.d.
Vistonis 7/2014	n.d.	n.d.	n.d.	n.d.	n.d.	n.d.	n.d.	n.d.	n.d.	n.d.	n.d.	n.d.	n.d.	n.d.	n.d.	n.d.	n.d.
**Mean**	**52**.**4**	**n**.**d**.	**n**.**d**.	**0**.**058**	**6**.**14**	**62**.**1**	**n**.**d**.	**22**.**0**	**n**.**d**.	**4**.**13**	**69**.**3**	**4**.**32**	**0**.**507**	**0**.**537**	**9**.**06**	**n**.**d**.	**n**.**d**.
**Median**	**52**.**4**	**n**.**d**.	**n**.**d**.	**0**.**058**	**1**.**96**	**36**.**5**	**n**.**d**.	**12**.**9**	**n**.**d**.	**0**.**076**	**21**.**7**	**0**.**58**	**0**.**507**	**0**.**537**	**9**.**06**	**n**.**d**.	**n**.**d**.

n.d. not detected.

In Lake Pamvotis several MC variants (dmMC-RR, MC-RR, MC-YR, MC-LR) were detected in water samples at relatively low concentrations (MC-LR at 131 μg L^−1^). The filtered biomass collected from the same sample (10/2014) contained only MC-RR and MC-LR at low concentrations, indicating the presence of a late bloom in the lake with progressive cell lysis, releasing CTs to the aqueous environment. In the past, extracellular TMCs were detected ranging from <1 μg L^−1^ eq. to 9 μg L^−1^ eq.^83^, 0.01–7.88 μg MC-LR eq. L^−1^ ^54^, 0.01 μg L^−1^−19.5 μg L^−1^ ^58^ and 0.310–2.4 μg L^−1^ ^52^, all of them using ELISA.

Lake Vegoritis (7/2014) contained extracellular dmMC-RR, MC-RR, MC-YR, dm^3^MC-LR, MC-LR, MC-LY (MC-RR at 104 μg L^−1^). In filtered water from Lake Mikri Prespa, MC-RR, MC-YR and MC-LR were detected. These were also detected in the filtered biomass of the sample. To the best of our knowledge, this is the first report of extracellular MCs, including their individual variants, in water from these two lakes, using LC-MS/MS.

Extracellular STX was detected in the Lake Kastoria during October 2014 (52.4 μg L^−1^). ANA-a was present only in one water sample (Lake Kastoria, September 2016) in trace concentration 0.058 μg L^−1^. This is the first report on the presence of extracellular STXs and ANA-a in Lake Kastoria, using LC-MS/MS. In the past, only one report of its presence in a Greek lake has been reported, using LC-MS/MS^85^ without stating the origin of the sample.

In the sample from Marathonas water reservoir (9/2013) no CTs were detected. The reservoir was studied thoroughly in the past by our group. Contrary to the present results, the lake water contained detectable extracellular MC concentrations. During 2007–2010, extracellular MC-YR, MC-LR and MC-RR were identified and varied seasonally, with maximum concentrations of 717, 451 and 174 ng L^−1^, respectively^69^. The same MC variants were also detected in Marathonas water reservoir during another study of our group using LC-MS/MS, but their concentrations were very low, ranging from 5–60 ng L^−1^ for MC-RR, 4–44 ng L^−1^ for MC-LR and <LOQ − 4 ng L^−1^ for MC-YR^60^.

### The case of Lake Kastoria

**Figure 3:**
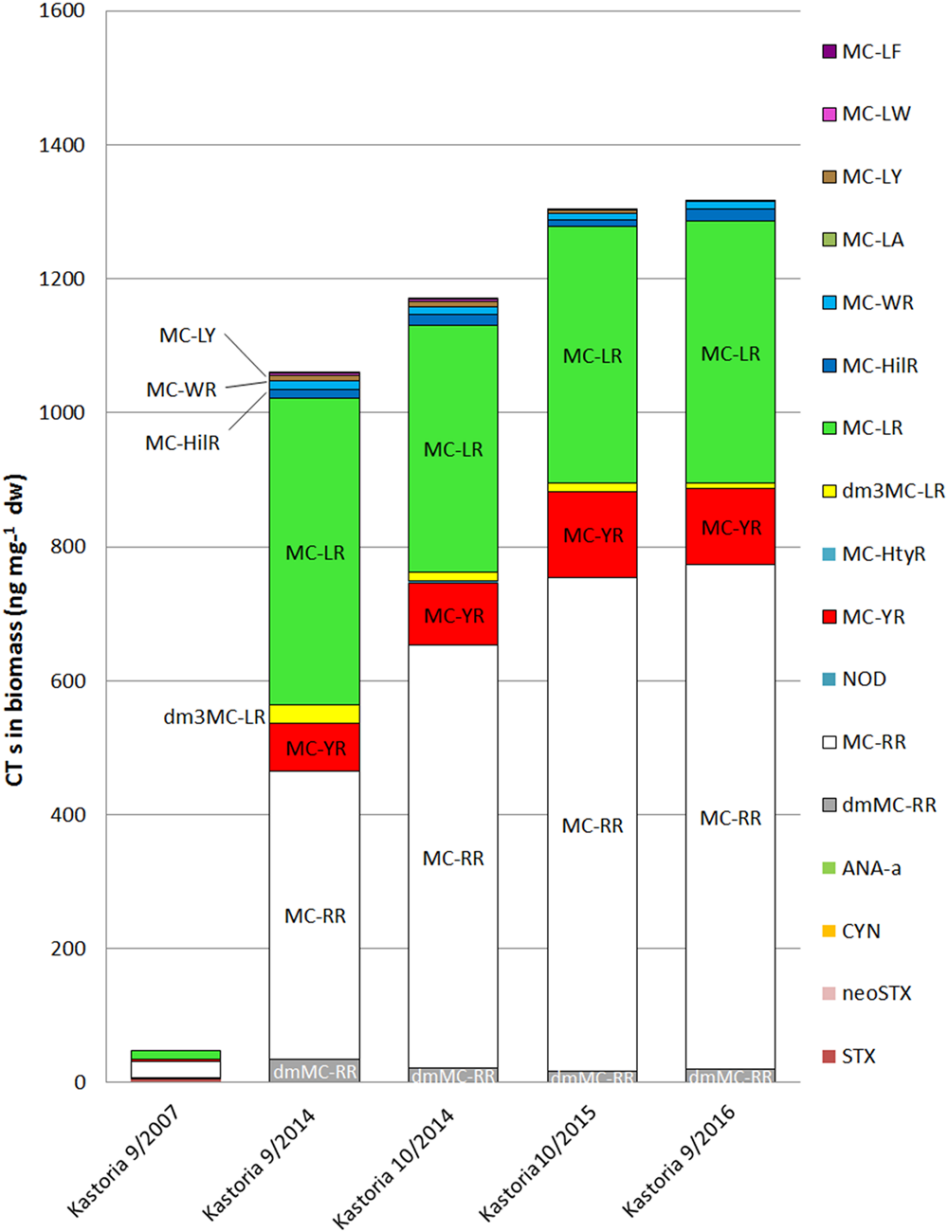
Diversity and content of cyanotoxins found in cyanobacteria obtained from Lake Kastoria.

In the case of Lake Kastoria, a series of samples were collected in different time periods, during the years 2007, 2014, 2015 and 2016. The analytical results reveal the consistent presence, increased diversity and abundance of various CTs in the biomass and filtered water of the lake, throughout the sampled periods (Fig. 3).

All the targeted toxins, except for CYN, ANA-a and neoSTX, have been identified throughout the different sampling periods in the biomass of the lake. MCs were the dominant CT group (Tables 1 and 2). The main MC present was MC-RR with a mean content of 516 ng mg^−1^ dw or 51.1 μg L^−1^ (range 24.7–754 ng mg^−1^ dw). The median content was 632 ng mg^−1^ or 63.2 μg L^−1^ (ranges in Fig. S4). MC-LR also occurred at high content levels (13.4–458 ng mg^−1^ dw) with mean and median values, 323 and 382 ng mg^−1^, or 32.0 and 38.2 μg L^−1^, respectively. MC-YR was present at increased levels (2.77–128 ng mg^−1^ dw) or 0.045–12.8 μg L^−1^. The biomass samples from Lake Kastoria also contained significant amounts of the desmethyl variants dmMC-RR and dm^3^MC-LR with mean content of 18.42 and 15.43 ng mg^−1^ dw, respectively. MC-WR, MC-HtyR, MC-LA, MC-LY, MC-LW and MC-LF have also been identified. The TMCs content in the case of Lake Kastoria ranged from 41.4–1316 ng mg^−1^ dw with a mean of 978.2 ng mg^−1^ dw or 97.1 μg L^−1^.

Apart from MCs, other CTs such as STX was detected in the biomass of Lake Kastoria, in the samples of September 2007 (6.10 ng mg^−1^ dw) and September 2014 (1.40 ng mg^−1^ dw). CYN and ANA-a were not detected in the biomass of lake Kastoria, although the presence of ANA-a was detected at trace concentration in filtered water during September 2016 (Table 4).

Regarding the extracellular MCs determined in the filtered water from Lake Kastoria, the main MC variant found was MC-RR, at concentrations ranging from 0.092 to 338 μg L^−1^ (ranges in Fig. S5). The mean concentration of the toxin was 75.0 μg L^−1^ and the median was 24.4 μg L^−1^. MC-LR was also present consistently in all samples at concentrations ranging from 0.072 to 354 μg L^−1^. The mean and median concentrations of MC-LR are 66.7 and 14.0 μg L^−1^, respectively. MC-YR was present at concentrations ranging 0.013–80.7 μg L^−1^. The desmethyl forms dmMC-RR and dm^3^MC-LR were present at lower concentrations (Table 4).

### Ratio of concentrations (intracellular MC-LR/MC-RR)

The ratio of MC-LR/MC-RR is known to change in relation to the available total phosphorus concentrations, light intensity and NO_3_-N concentration^86^. The effect of various environmental parameters on the ratio of the produced MC congeners and their relative abundances has been evaluated by a few studies, suggesting that the cellular composition of MC variants may change in response to changing environmental conditions, such as temperature^87^, light intensity and nutrient supply^88^, photon irradiance^47^ or amino acid availability (leucine and arginine)^89^. The ratio of different MC-congeners is strongly related to the dominant environmental parameters of a surface water body, the dominant cyanobacterial species and their growth stage. Nevertheless, the precise mechanisms determining the composition of MC variants in cyanobacteria are still debated^47^. The toxicity of MC-LR is far higher than that of MC-RR^90^, therefore low values of MC-LR/MC-RR ratios in combination with low TMC concentrations are desirable.

**Figure 4:**
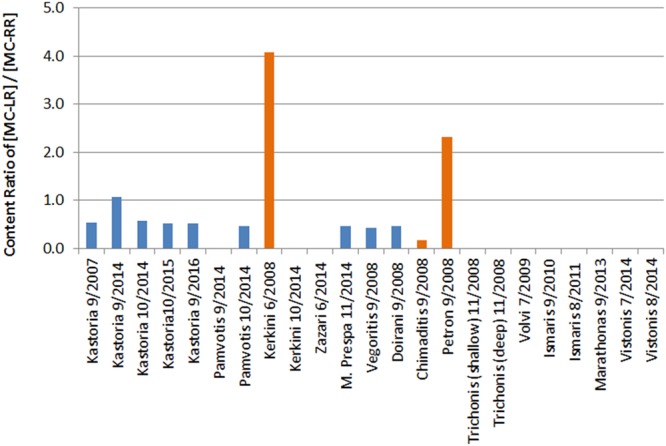
MC-LR/MC-RR content ratios in different biomass samples from Greek lakes (samples divided in two groups with different colors).

Content ratios of two common MCs (intracellular MC-LR/MC-RR) were calculated for Lake Kastoria samples, collected throughout the sampling periods and found to range from 0.5 to 1.1 (Fig. 4). The situation was similar in other Greek lakes (Pamvotis, Mikri Prespa, Vegoritis and Doirani), where both these MC variants were detected. The production of certain MC variants and their ratio is largely related to the toxin producing cyanobacterial species present in the sample. Kastoria was mainly dominated by *Microcystis aeruginosa*, *Microcystis panniformis*, *Anabaena cf*. *flos*-*aquae*, *Anabaena cf*. *circinalis* throughout the years of sampling with *Cylindrospermopsis raciborskii* dominant in 2007 (Table 1). Lakes Pamvotis and Mikri Prespa presented similar dominant cyanobacterial species (Pamvotis: *Microcystis aeruginosa*, *Anabaena flos*-*aquae*, Mikri Prespa: *Microcystis aeruginosa)*, (Lake Vegoritis:*Anabaena viguieri*, *Aphanizomenon flos*-*aquae*, *Anabaena flos*-*aquae* and *Microcystis aeruginosa*). In Lake Doirani the dominant species were *Cylindrospermopsis raciborskii*, *Raphidiopsis mediterranea*, *Microcystis aeruginosa*, *Aphanizomenon issatschenkoi*, *Aphanizomenon gracile*, *Anabaena flos*-*aquae* and *Anabaena aphanizomenoides*).

In contrast, Lakes Kerkini (2008), Chimaditis and Petron presented different ratios (4.1, 0.2 and 2.3, respectively), shown in different colour in Fig. 4, which was accompanied by more diverse cyanobacterial assemblancies (Table 1). The highest intracellular MC-LR/MC-RR ratio values were recorded in lakes Kerkini and Petron and coincided with the highest cyanobacterial species diversity per sample. More specifically, in Lake Kerkini the dominant cyanobacteria were *Aphanizomenon flos*-*aquae*, *Microcystis aeruginosa*, *Microcystis panniformis* and several species of Nostocales (*Cylindrospermopsis raciborskii*, *Anabaena* cf. *viguieri*, *Anabaenopsis elenkinii*, *Anabaena flos*-*aquae*, *Anabaena aphanizomenoides*) and Oscillatoriales (*Pseudanabaena limnetica*) (Fig. S6), while Lake Chimaditis was dominated by *Microcystis panniformis*, *Microcystis aeruginosa*, *Microcystis wesenbergii*, *Microcystis flos*-*aquae*, *Cylindrospermopsis raciborskii* and *Anabaena flos*-*aquae*. Finally, Lake Petron was dominated by *Planktothrix agardhii*, *Cylindrospermopsis raciborskii*, *Cyanodictyon imperfectum*, *Aphanizomenon gracile*, *Anabaenopsis elenkinii*, *Planktolyngbya limnetica* and *Planktolyngbya microspira* (Fig. S7). The differences in cyanobacterial diversity could possibly explain the variation of MC-LR/MC-RR content ratios of these lakes. Also, light conditions in shallow lakes have been known to influence certain cyanobacterial species, e.g. *Planktothrix agardhii*, is enhanced at higher irradiance conditions. This species is known to produce less arginine-based MCs (MC-RR) and more leucine-based MCs^47^. This could possibly explain the different content ratios in a shallow lake, such as Lake Petron.

In the past, Gkelis *et al*.^55^, have reported MC-LR/MC-RR ratios in several Greek lakes dominated by *Microcystis* ranging from 0.4 to 10, which is in accordance with our present findings. The ratio of MC-LR/MC-RR under controlled laboratory cultures of *Microcystis aeruginosa UTEX 2388*, has been reported to range approx. 0.2–0.5^91^. The intracellular MC-LR/MC-RR ratios, observed in mixed cyanobacterial communities dominated by *Aphanizomenon aphanizomenoides*, *Microcystis wesenbergii*, *Limnothrix redekei*, were reported to range approx. 0.5–3.0 and this is close to the values reported in the present study.

### Risk Assessment for water intended for recreational use

The World Health Organization (WHO) has set guideline values for cyanobacteria and MCs present in waters used for recreational purposes^41^. Three guideline levels have been established, a guideline value of 2–10 μg L^−1^ of TMCs, corresponds to low probability of adverse health effects, alerting the authorities in order to initiate further surveillance of the site. TMCs concentration of 10–20 μg L^−1^ (if *Microcystis* sp. dominates the bloom), corresponds to moderate probability of adverse health effects, triggering further action and daily inspection of scum formation by the authorities. TMCs values higher than 20 μg L^−1^ (if *Microcystis* dominate) correspond to high probability of adverse health effects. Guidance values have also been issued by the Oregon Public Health Division (OPHD) for MCs, CYN and ANA-a and are shown in Table S5^42,92,93^. The TΜCs found in the samples of the present study were compared to the guidance values established by WHO.

**Figure 5:**
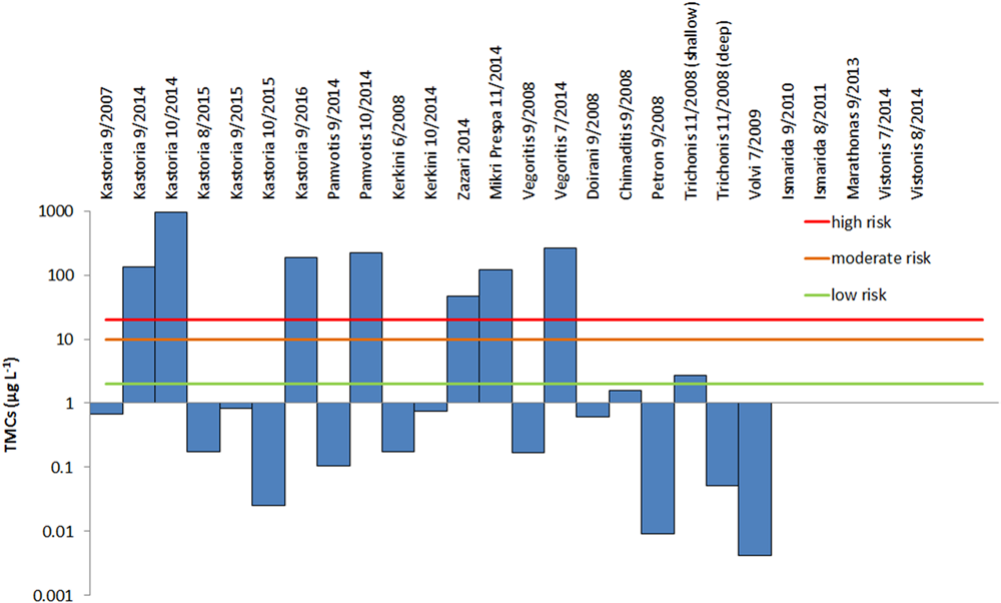
Total MC Concentration TMC (sum of intracellular and intracellular MCs) in Greek lakes (logarithmic scale). The potential risk of adverse health effects rising from the presence of MCs in lakes is indicated based on the established WHO guidelines.

Based on the probability of adverse health effects of the TMC content, each lake sample was categorized according to the classification proposed by WHO (Table S5). Figure 5 shows that seven of the analyzed samples contained MCs at a concentration that poses a high risk of adverse health effects, including lakes Kastoria, Pamvotis, Zazari, Mikri Prespa and Vegoritis. The sample from Lake Trichonis posed low to moderate risk of inducing adverse health effects.

Regulations for the presence of CTs in surface and drinking water nowadays, mainly accept the oral route (ingestion of toxins via drinking-water, recreation or consumption of fish) as the main vehicle of CT exposure^39,40,42^. The Tolerable Daily Intake (TDI) for the average adult or child, is described as the amount of a potentially harmful substance (in this case MCs) that can be consumed daily over a lifetime, with negligible risk of adverse health effects^14^. WHO derived that the TDI of MCs for humans should be 0.04 μg kg^−1^ bw^43,92,93^. TDI values for other CTs have also been set, e.g. by the OPHD which has also set TDI values for CYN, ANA-a and TMCs (0.03, 0.1 and 0.05 μg kg^−1^ bw, respectively)^42^ (Table S5).

Table 5 shows the minimum volume of surface water (mL) that has to be accidentally consumed by a child or adult swimmer at the sampling points, in order to reach the tolerable daily intake TDI set by WHO and OPHD. In Lake Kastoria during 2014, as low as 2.55 mL of lake water was enough to reach the TDI (for MCs) by an adult swimmer and only 0.42 mL by a child. Similar is the case of lakes Pamvotis, Zazari, Mikri Prespa and Vegoritis, where consumption of only a few mL is enough to reach the maximum TDI for MCs. This means that if an average adult swimmer accidentally consumes 200 mL of lake water in the sampling point, then the water of Lake Kastoria (10/2014) would provide 7859% of the TDI (Table 6). These values indicate the risk associated to the use of this water for daily recreational activities.

**Table 5:** Volume (mL) to be accidentally consumed in order to reach the amount of tolerable daily intake (TDI) for each toxin.

Sample	Adults			Children		
	CYN	ANA-a	TMCs	CYN	ANA-a	TMCs
Kastoria 9/2007			3541			590
Kastoria 9/2014			18.1			3.01
Kastoria 10/2014			2.55			0.42
Kastoria 8/2015			13559			2260
Kastoria 9/2015			2909			485
Kastoria 10/2015			10.8			1.84
Kastoria 9/2016			12.7			1.84
Pamvotis 9/2014	276748		22634	46100		3772
Pamvotis 10/2014			10.7			1.79
Kerkini 6/2008		9892	13635		1650	2273
Kerkini 10/2014			3255			543
Zazari 6/2014			51.2			8.53
Mikri Prespa 11/2014			19.6			3.27
Vegoritis 9/2008	325439		14369	54200		2395
Vegoritis 7/2014			9.13			1.52
Doirani 9/2008	73967	6477	3933	12300	1080	655.5
Chimaditis 9/2008			1546			258
Petron 9/2008			262640			43773
Trichonis 11/2008 (shallow)			888			148
Trichonis 11/2008 (deep)			46306			7718
Volvi 7/2009			582289			97048
Ismarida 9/2010						
Ismarida 8/2011						
Marathonas 9/2013						
Vistonis 7/2014						
Vistonis 8/2014

**Table 6:** Percentage % of TDI reached when accidentally consuming water (200 mL).

Sample	Adults			Children		
	CYN	ANA-a	TMCs	CYN	ANA-a	TMCs
Kastoria 9/2007			5.65			33.9
Kastoria 9/2014			1107			6644
Kastoria 10/2014			7859			47154
Kastoria 8/2015			1.48			8.85
Kastoria 9/2015			6.88			41.3
Kastoria 10/2015			1817			10901
Kastoria 9/2016			1568			9406
Pamvotis 9/2014	0.072		0.884	0.434		5.30
Pamvotis 10/2014			1863			11180
Kerkini 6/2008		2.02	1.48		12.1	8.88
Kerkini 10/2014			6.14			36.9
Zazari 6/2014			391			2344
Mikri Prespa 11/2014			1020			6119
Vegoritis 9/2008	0.061		1.39	0.369		8.35
Vegoritis 7/2014			2192			13149
Doirani 9/2008	0.270	3.09	5.08	1.62	18.5	30.5
Chimaditis 9/2008			13.1			78.6
Petron 9/2008			0.076			0.457
Trichonis 11/2008 (shallow)			22.5			135
Trichonis 11/2008 (deep)			0.446			2.68
Volvi 7/2009			0.034			0.206
Ismarida 9/2010						
Ismarida 8/2011						
Marathonas 9/2013						
Vistonis 7/2014						
Vistonis 8/2014						

### Diversity of Cyanobacteria in Greek lakes

Microscopic analysis revealed the diversity and abundance of cyanobacteria in Greek lakes throughout the course of the study (Figures S6 and S7). In total, 55 different cyanobacterial species were identified, originating from the three main orders (Chroococcales, Oscillatoriales and Nostocales) with representatives in the plankton, thoroughly described in Table 1.

The largest diversity of cyanobacterial species was found in the shallow Lake Petron (20 different species), followed by Kerkini Reservoir (19 species) and Lake Chimaditis (13 species), while the lowest diversity were observed in Lake Mikri Prespa (3 species) and Lake Vistonis (3 species). One of the most commonly occurring cyanobacterial genus was *Microcystis* (*M*. *aeruginosa*, *M*. *panniformis*, *M*. *wesenbergii*, *M*. *flos*-*aquae*), which dominated most of the Greek lakes and has been linked to the production of various CTs, mainly MCs^10,20,94^. Although various cyanobacterial species were simultaneously found to co-occur in most of the studied lakes, only a few species (less than 5) dominated (contributing to >10% of biomass) in each lake.

#### MCs in relation to cyanobacteria species

Studies have shown that there are significant associations between specific MC congeners and particular cyanobacteria species. The relative biomass of *Microcystis aeruginosa* has been associated with MC-RR, *Microcystis wesenbergii* with MC-LA and *Aphanizomenon flos*-*aquae* with MC-YR^95,96^.

The most commonly found CTs in the Greek lakes were MCs. The production of MCs has been reported in the past by cyanobacterial species of the genera *Anabaena*, *Anabaenopsis*, *Aphanocapsa*, *Aphanizomenon*, *Cylindrospermopsis*, *Fischerella*, *Hapalosiphon*, *Lyngbya*, *Microcystis*, *Nostoc*, *Oscillatoria*, *Planktothrix*, *Phormidum*, *Rivularia*, *Synechococcus* and *Arthrospira* (*A*. *fusiformis*), although the main genera reported to be responsible for their production are *Anabaena* and *Microcystis*^20^. In the majority of the studied Greek lakes, the production of MCs seems to be mainly related to the occurrence of *Microcystis*, *Anabaena* and *Planktothrix*.

More specifically, in Lake Kastoria, blooms were dominated by *Μicrocystis aeruginosa*, *Microcystis panniformis* as well as *Anabaena* (e.g. *A*. *flos*-*aquae*) which could be responsible for the production of the highly diverse and abundant CT content (Table 1). The co-existence of various other cyanobacterial species, could have contributed to the increased production of MCs as well as STX. The dominant cyanobacterial species however belonged to *Microcystis* and *Anabaena* genera, which are expected to produce a high diversity of MC variants. However, the analysis of biomass samples including mixed cyanobacterial species, does not allow the safe discrimination and univocal correlation of the species responsible for the production of each CT.

Lake Doirani also presents a high diversity of MC variants (Fig. 1a) as well as CYN and ANA-a. Cyanobacterial species *Μicrocystis aeruginosa*, *Anabanena flos*-*aquae*, *Α*. *aphanizomenoides* were dominant in the lake bloom, while *Cylinrospermopsis raciborskii*, *Cuspidothrix (Aphanizomenon) issatschenkoi* and *Aphanizomenon gracile* were also found in lower biovolume. These species’ association could be responsible for the diversity of CTs present in the bloom (Table 1).

In Lake Chimaditis, a wide variety of MCs was observed (dmMC-RR, MC-RR, MC-YR, MC-LR, MC-HilR). The species *Μicrocystis aeruginosa*, *Microcystis panniformis* and *Microcystis flos*-*aquae* were dominant in the sample and possibly contribute to the production of MCs, while *Cylindrospermopsis raciborskii*, *Aphanizomenon issatschenkoi* and *Anabaena flos*-*aquae* were also present in lower quantities. The occurrence of *Merismopedia* could also contribute to the production of various MCs^97^.

In Lake Petron, only MC-RR and MC-LR were identified, although the lake water presented the highest cyanobacterial diversity. MC- producing species such as *Μicrocystis aeruginosa*, *Microcystis panniformis* and *Microcystis flos*-*aquae* were absent from the lake bloom. *Microcystis wesenbergii* contributed to the cyanobacterial biomass, although it has been reported as a non-producing MC species^96^. *Cylindrospermopsis raciborskii*, *Cyanodictyon imperfectum*, *Planktolyngbya limnetica*, *Planktolyngbya circumcreta* and *Aphanizomenon gracile* were found to be the dominant species (Fig. S7). In the past *Cylindrospermopsis raciborskii*, *Aphanizomenon*, *Anabaenopsis*, *Planktothrix* as well as *Aphanocapsa* and *Merismopedia* have been related to the production of several MCs^97^.

In Lake Vegoritis, MC-RR and MC-LR as well as CYN, were detected, while the dominant species was *Μicrocystis aeruginosa* (Table 1). *Aphanizomenon* and *Anabaena* were also present in the bloom.

Desmethyl MCs have been reported in the past as the main MC variants produced during blooms dominated from *Planktothrix* and *Microcystis*^80^. In lake Trichonis, a characteristic bloom of *Planktothrix rubescens* in deep water (23–40 m) was observed, while 2 out of 3 MC-variants identified in the bloom were desmethyl MCs (Table 1). *Planktothrix rubescens* has been known to produce [D-Asp^3^]MCs^98^, which is in agreement with the present study.

In Lake Volvi, only *Nostocales* taxa were observed (*Anabaena*, *Aphanizomenon* and *Cylindrospermopsis)*. The occurrence of these species is not necessarily related to the production of MCs^99^. *Anabaenopsis* was also present in the sample and has been linked to the production of MCs^100^, Ιn Lake Ismaris, the dominant cyanobacterial species were also *Anabaena*, *Anabaenopsis*, *Aphanizomenon*, *Cylindrospermopsis* and additionally *Planktothrix*. No MCs were observed, although bloom biomass levels were recorded (53 mg L^−1^ of cyanobacteria).

The dominance of *Μicrocystis aeruginosa* (Table 1) in the bloom of Lake Zazari was accompanied by the presence of MC-RR and MC-YR. Also present in the lake were *Microcystis wesenbergii*, which is known as a MC non-producting species, *Anabaena spiroides* which is also known for its non-toxic strains^101^ and *Merismopedia warmingiana*^97^.

*Μicrocystis aeruginosa*, *Microcystis panniformis* as well as *Anabaena*, *Aphanizomenon* and *Planktothrix* were identified in Lake Pamvotis, which are able to produce multiple MC-variants. LC-MS/MS analysis revealed the presence of MC-RR, MC-YR and MC-LR in the biomass.

Similarly, in Lake Mikri Prespa, *Microcystis aeruginosa*, *Anabaena* cf. *lemmermanii* and *Aphanocapsa* sp. were the dominant species, with dmMC-RR, MC-RR and MC-LR identified in the sampled biomass.

No MCs were identified in Lake Vistonis, where *Αphanizomenon favaloroi*, *Pseudanabaena limnetica* and *Limnothrix* sp., were the most abundant species. This is in accordance with past studies in Mediterranean lakes, where the production of MCs is mainly linked to the presence of *Microcystis*. In a recent study in Spanish lakes, 31 Nostocales strains were isolated and analyzed with negative results for MCs^99^.

In Lake Kerkini, during June 2008, MC-RR, MC-LR and MC-LW were identified in the biomass, which mainly contained the MC-producing cyanobacterial species *Μicrocystis aeruginosa*, *Microcystis panniformis*, and *Anabaena* (Table 1), as well as other cyanobacterial species known to produce MCs, such as *Cylindrospermopsis raciborskii*, *Planktothrix agardhii* and several *Aphanizomenon* species (Fig. S8).The production of the identified MCs cannot be safely attributed to these species. It is noteworthy, that the cyanobacterial bloom, which was sampled from the same lake during October 2014, showed no evidence of MC content. The sample contained mainly *Cylinrospermopsis raciborskii* and *Planktothrix agardhii*. No *Microcystis* species were identified.

#### ANA-a in relation to species

ANA-a was identified only in Lake Doirani (September 2008, 65.5 ng mg^−1^dw) and Lake Kerkini (June 2008, 61.7 ng mg^−1^dw) as shown in Table 2. The production of ANA-a has been reported in the past by the following cyanobacterial species: *Anabaena* spp., *Anabaena flos*-*aquae*, *Aphanizomenon flos*-*aquae*, *Cylindrospermum* sp., *Anabaena planctonica*, *Anabaena crassa*, *Planktothrix rubescens*, *Raphidiopsis mediterranea*, *Phormidium favosum*, *Aphanizomenon issatschenkoi*, *Αrthrospira fusiformis* and *Phormidium autumnale*^102^. The bloom from lake Doirani (September 2008) was dominated by species *Aphanizomenon issatschenkoi*, *Anabaena flos*-*aquae*, *Aphanizomenon flos*-*aquae*, *Raphidiopsis mediterranea* and *Cylindrospermopsis raciborskii*, directly related to the production of ANA-a. The sample from Kerkini Reservoir (June 2008) contained mainly *Aphanizomenon flos*-*aquae*, *Microcystis aeruginosa*, *Cylindrospermopsis raciborskii*, *Anabaena* cf. *viguieri*, *Anabaenopsis selenkinii*, *Anabaena flos*-*aquae*, *Microcystis panniformis*, *Anabaena aphanizomenoides*, some of which are closely related to the production of ANA-a. Water samples from these two lakes did not contain detectable amounts of ANA-a. It is noteworthy, that although several other bloom samples from Greek lakes in this study contained some of those ANA-a producing cyanobacterial species, their analysis indicated negative results for the presence of ANA-a.

#### CYN in relation to species

CYN was identified in trace levels in the biomass of lakes Doirani (September 2008, 1.7 ng mg^−1^ dw), Vegoritis (September 2008, trace amount) and Pamvotis (September 2014, trace amount) as shown in Table 4. In the past the production of CYN has been related to species such as *Cylindrospermopsis raciborskii*, *Aphanizomenon ovalisporum* (synonym *Anabaena bergii*), *Aphanizomenon flos*-*aquae*, *Anabaena lapponica*, *Raphidiopsis curvata*, *Umezakia natans*, *Αphanizomenon gracile* and *Lyngbya wollei*^30^. In the case of Lake Doirani, CYN could be related to the dominance of *Cylindrospermopsis raciborskii*/*Raphidiopsis mediterranea* as well as *Aphanizomenon gracile*, while in lakes Vegoritis and Pamvotis, the occurrence of CYN in trace amounts could be related to the presence of *Aphanizomenon flos*-*aquae*, which usually forms blooms in those lakes during the summer period. Although *Cylindrospermopsis raciborskii*/*Raphidiopsis mediterrane* is usually abundant in Lake Vegoritis throughout various periods, it was not detected during the specific sampling period.

#### STX and neoSTX in relation to species

STX was identified in the bloom samples from Lakes Kastoria (2007, 2014), Kerkini (2008, 2014), Petron (2008), Trichonis (2008) and Vistonis (2014). In the samples from Kerkini (2008), Trichonis (2008) and Vistonis (2014), neoSTX was also found to be present. STX and neoSTX production has been linked in the past with *Αphanizomenon flos*-*aquae*, *Planktothrix* sp., *Anabaena circinalis*, *Anabaena lemmermannii*, *Aphanizomenon* sp., *Aphanizomenon gracile*, *Cylindrospermopsis raciborskii* and *Lyngbya wollei*^38,48,49^.

In Lake Kastoria, the production of STX could be attributed to the presence of cyanobacteria *Anabaena* cf. *circinalis*, while in lakes Petron, Trichonis and Kerkini, the presence of STX concurs with the occurrence of cyanobacterial blooms dominated by *Aphanizomenon gracile*/*Cylindrospermopsis raciborskii* (Lake Petron), *Αphanizomenon flos*-*aquae* (Lake Trichonis) and *Cylindrospermopsis raciborskii*/*Αphanizomenon flos*-*aquae* (Kerkini Reservoir). In those water bodies, other cyanobacterial species could also be potentially responsible for the production of STX, like *Planktothrix* (*P*. *agardhii* and *P*. *rubescens*). Especially for Lake Trichonis, STX was detected in the bloom dominated by *P*. *rubescens*.

## Conclusions

A wide variety of intracellular CTs was identified in the biomass from Greek lakes, including 12 MCs (dmMC-RR, MC-RR, MC-YR, MC-HtyR, dm^3^MC-LR, MC-LR, MC-HilR, MC-WR, MC-LA, MC-LY, MC-LW, MC-LF), CYN, ANA-a, STX and neoSTX. This wide diversity of CT groups and MC-variants is reported for the first time in the cyanobacterial biomass of Greek lakes. The results were obtained with the application of a comprehensive analytical workflow using a collection of definitive analytical methods based on LC-MS/MS.

The most commonly found CT group in the cyanobacterial biomass of Greek lakes was MCs, whereas the most common cyanobacteria were species of the genera *Microcystis* and *Anabaena*. MC-RR was the most frequently detected MC-variant, followed by MC-LR, MC-YR and dmMC-RR. Lake Kastoria, presented the highest variety of CTs, with 12 MC-variants and STX, which is the most diverse cocktail of toxins identified in Greece. Intracellular MC-RR was the most abundant toxin, reaching 754 ng mg^−1^ dw. MC-LR was found at intracellular content up to 458 ng mg^−1^ dw.

Lake Doirani also presented a large diversity of toxins in the cyanobacterial biomass, including CYN, ANA-a and 4 MCs (dmMC-RR, MC-RR, MC-YR, MC-LR). Lake Trichonis presented biomass samples with 3 MCs (dmMC-RR, MC-YR, dm^3^MC-LR), STX and neoSTX. In Lake Kerkini 3 MCs (MC-RR, MC-LR, MC-LW), ANA-a, STX and neo-STX were identified. In all other lakes a smaller number of toxins was identified. Lake Trichonis in November 2008, contained only the desmethyl variants dmMC-RR and dm^3^MC-LR.

CYN was identified for the first time in the biomass of lakes Pamvotis, Vegoritis and Doirani at trace concentrations. ANA-a was detected in lakes Kerkini (6/2008) and Doirani (9/2008). These are the first reports of ANA-a presence in specific Greek lakes using LC-MS/MS. STX and neo-STX were identified for the first time in biomass samples from lakes Trichonis, Vistonis and Petron.

The analysis of filtered water indicated the presence of various extracellular CTs, mainly MCs (dmMC-RR, MC-RR, MC-YR, dm^3^MC-LR, MC-LR, MC-HilR, MC-LY) as well as ANA-a and STX. Lake Kastoria presented the highest number and diversity of CTs in nearly all the water samples, with 8 different MCs (dmMC-RR, MC-RR, MC-YR, dm^3^MC-LR, MC-LR, MC-HilR, MC-WR, MC-LA). Lake Vegoritis contained extracellular dmMC-RR, MC-RR, MC-YR, dm^3^MC-LR, MC-LR, MC-LY, while Lake Pamvotis contained extracellular dmMC-RR, MC-RR, MC-YR, MC-LR. Extracellular STX was detected in the Lake Kastoria during October 2014 and ANA-a was present only in one water sample (Lake Kastoria, September 2016) at trace concentration.

The concentration ratios of two common MCs (intracellular MC-LR/MC-RR) in all the samples of Lake Kastoria, throughout the sampling periods, ranged from 0.5 to 1.1. The situation was similar in other Greek lakes (Pamvotis, Mikri Prespa, Vegoritis and Doirani), where both these MC variants were detected. All these lakes showed the presence of related toxin-producing cyanobacteria species, i.e. *Microcystis aeruginosa* and *Anabaena flos*-*aquae*. In contrast, Lakes Kerkini (2008), Chimaditis and Petron presented different ratios (4.1, 0.2 and 2.3, respectively), which was accompanied by more diverse cyanobacterial assemblages. The highest intracellular MC-LR/MC-RR ratio values were recorded in lakes Kerkini and Petron and coincided with the highest cyanobacterial species diversity per sample.

Seven of the analyzed samples from Greek lakes (Kastoria, Pamvotis, Zazari, Mikri Prespa and Vegoritis), contained MCs at a concentration that poses a high risk of inducing adverse health effects, according to WHO guidelines. In those lakes, the accidental consumption of only a few milliliters of lake water by an average adult swimmer would be enough to reach the TDI set for MCs.

## Materials and Methods

### Chemicals and reagents

Standards of MC variants [D-Asp^3^]MC-LR, [D-Asp^3^]MC-RR, MC-WR, MC-HtyR, MC-HilR, MC-LY, MC-LW and MC-LF were purchased from ENZO Life Science (Lausen, Switzerland). [D-Asp^3^]MC-LR standard was used for the identification and quantification of demethylated MC-LR isomers in position (3) and were called dm^3^MC-LR. The standard [D-Asp^3^]MC-RR was used for demethylated MC-RR variants in positions (3) and (5) and were called dmMC-RR (further information is included in the end of the supplementary information). MC-RR, MC-LR, MC-YR, and MC-LA standards were supplied by Sigma-Aldrich (Steinheim, Germany). CYN was purchased from Abraxis (Warminster, UK) and a racemic mixture of (±  ANA-a Fumarate from TOCRIS Bioscience (Bristol, UK). ANA-a concentration was calculated based on the exact concentration of fumarate, which was determined using high-purity standard solutions of fumaric acid, analyzed with a HPLC-UV system. Fumaric Acid (>99%) was provided by Sigma-Aldrich, Germany. Since most of the naturally occurring ANA-a is found in the (+) form^36^ and our method determines the total amount of ANA-a forms with the same ion transitions (*m/z*), co-eluting in the same retention time, then our choice of standard does not influence the analytical result. STX (NRC CRM-STX-e 65 μM) and neoSTX (NRC CRM-NEO-b 65 μM) were provided by NRC Canada. All substances had purity >95%. Methanol (MeOH) of HPLC grade (99.99%) was obtained from Fischer Scientific (Leics, UK), acetonitrile (ACN) of gradient grade for HPLC (≥99.9%) was obtained from Sigma-Aldrich (St. Louis, MO, USA) and n-Butanol (n-BuOH) 99.5% was obtained from Penta (Prague, Czech Republic). Dichloromethane (DCM) and Methyl tert-butyl ether (MTBE) were of analytical grade and obtained by Sigma-Aldrich (St. Louis, MO, USA). Formic acid (FA) (>98%) was purchased by Riedel-de Haën (Seelze, Germany). High purity water (18.2 MΩ cm) was produced on-site using a Temak TSDW10 system (Carlsbad. USA).

### Sample collection and preparation

**Figure 6:**
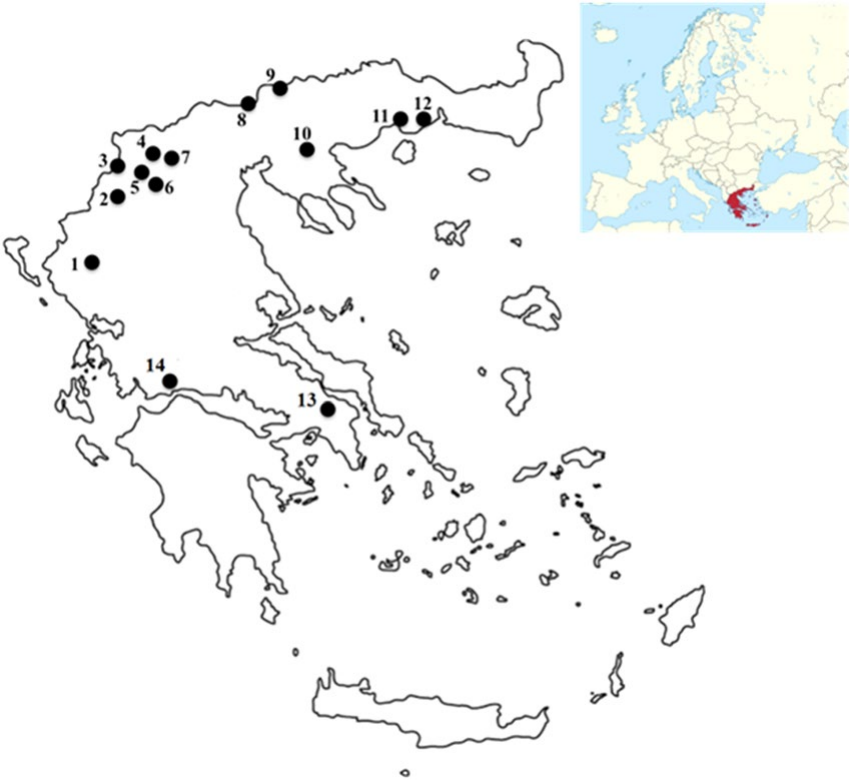
Map of Greece indicating the locations of the 14 water bodies (lakes and water reservoirs) examined for the occurrence of cyanobacteria and CTs: (1) Pamvotis, (2) Kastoria, (3) Mikri Prespa, (4) Petron, (5) Chimaditis, (6) Zazari, (7) Vegoritis, (8) Doirani, (9) Kerkini, (10) Volvi, (11) Vistonis, (12) Ismarida, (13) Marathonas, (14) Trichonis.

Water samples were collected from 14 different lakes and surface water reservoirs in Greece (Fig. 6) during 2007–2016. Samples were obtained from offshore sampling points from the euphotic zone (determined as 2.5xSecchi depth) using a 2 L Niskin type sampler. In Trichonis samples were collected from the lake deepest point both from shallow layer (12 m) and the deep water maxima of *Planktothrix rubescens* (23–40 m). In Lake Kastoria, additional surface samples were collected from inshore points in September 2014, October 2015 and September 2016. A sub-sample (500 mL) from each lake was transferred in a polyethylene bottle, where it was fixed with Lugol’s solution and with formaldehyde and it was used for microscopic analysis^103^. For CT analysis a part of each sample was kept in airtight polyethylene bottles and was transported to the laboratory for analysis in cooler containers at 4 °C^54,69,70^. Further preparation of the sample is described in section 4.4.

### Microscopic analysis

Water samples for microscopic analysis were collected. Fresh and preserved phytoplankton samples were examined under an inverted light microscope (Nikon TE 2000-S) and species were identified using taxonomic keys^104–107^. Phytoplankton counts (cells, colonies, and filaments) were performed using the Utermöhl’s sedimentation method^108,109^. For biomass estimation (mg L^−1^), the dimensions of 30 individuals (cells, filaments, or colonies) of each species were measured using tools of a digital microscope camera (Nikon DS-L1), while mean cell or filament volume estimates were calculated using appropriate geometric formulae^84^. Species and taxonomical groups comprising more than 10% (w/w) of the total phytoplankton biomass were considered to be dominant. The cell volume and total phytoplankton biovolume estimates were converted to biomass (wet weight) by assuming a density of 1 g cm^−3^.

### Sample treatment for CT analysis – Analytical workflow

**Figure 7:**
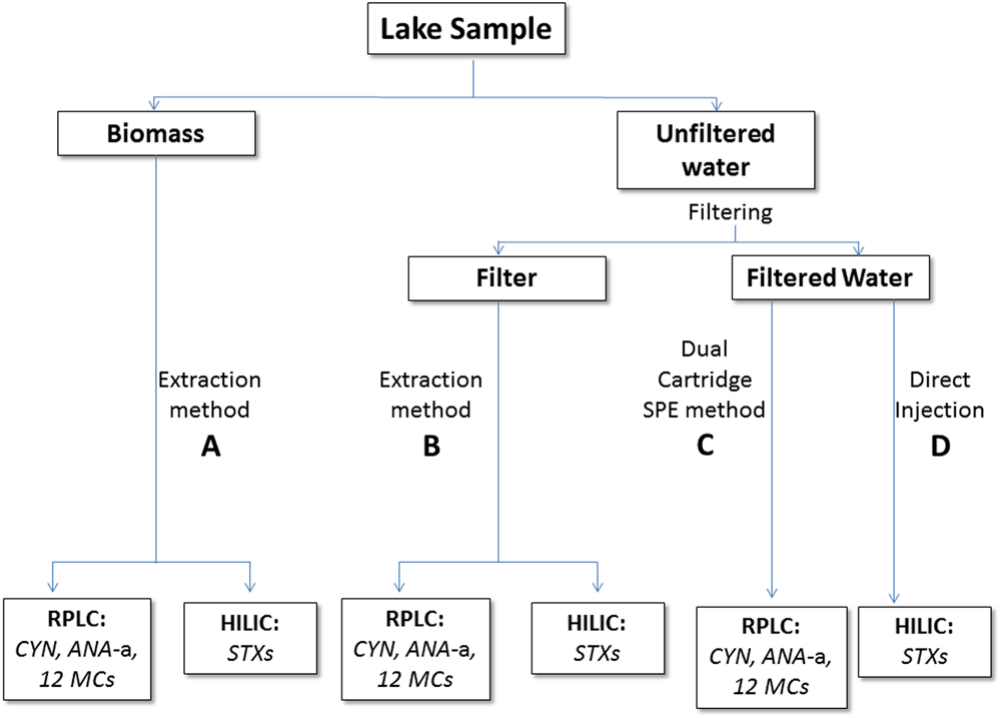
Workflow of the analytical methods used for the determination of CTs.

A detailed analytical workflow was developed including a complete set of the available validated methods for the analysis of CYN, ANA-a, 12 MCs, STX and neoSTX in various sample types (biomass, filters and filtered water). Four methods (A, B, C, D) were applied as following: (A) for CYN, ANA-a, 12 MCs and STXs in cyanobacterial biomass, (B) for CYN, ANA-a, 12 MCs, NOD and STXs in filters, (C) for CYN, ANA-a, 12 MCs in filtered water and (D) for STX and neoSTX in filtered water. A flow chart of the methods used for the determination of CTs is given in Fig. 7. All methods were validated in order to assess specificity, linearity, precision (repeatability and reproducibility), accuracy (% recovery), as well as limits of detection (LODs)/quantification (LOQs). To assess specificity, blank samples were analyzed and no interfering peaks were observed close to the retention times (t_R_) of the analytes.

The developed workflow provides a useful toolkit for the determination of various CTs in different matrices. After receiving the samples in the laboratory, alternative protocols were followed, according to the purpose and scope of the analysis. In cases where there was a surface bloom in the lake, cyanobacterial cells (biomass) were withdrawn from the surface layer of samples, using a glass pipette. The collected biomass was subsequently frozen and then transferred to a lyophilization apparatus (Martin Christ ALPHA 1–2, Vacuubrand HV Pump), where vacuum was applied for 24 h, at − 51 °C. Lyophilized biomass was further analyzed using ***method A***. If the samples did not contain visible biomass, they were filtered through GF/F filters (previously dried and pre-weighed). Filters where then extracted and analyzed with ***method B***. Selected filtrates from samples with known history of dissolved CTs were stored at 4 °C for further analysis of extracellular soluble CTs using ***methods C*** and ***D***.

A detailed description of the methods is given below:

#### Method A

Lyophilized biomass samples were extracted for the analysis of CYN, ANA-a, NOD and 12 MCs (dmMC-RR, MC-RR, MC-YR, MC-HtyR, dm^3^MC-LR, MC-LR, MC-HilR, MC-WR, MC-LA, MC-LY, MC-LW, MC-LF) using an optimized in-house method. The method was developed for the efficient extraction of target CTs from cyanobacterial biomass. Several parameters were assessed with the aim to maximize the extracted amount of toxins from biomass. For method optimization purposes, a lyophilized toxin-free biomass sample was spiked with a mixture of target CTs at a concentration level of 25 ng mg^−1^ dw and it was analyzed, in order to determine the optimum extraction conditions based on the obtained % recovery. The evaluated extraction parameters were: sonication type (probe, bath), sonication time (0–30 min), solvent types (H_2_O, MeOH, n-BuOH, DCM, MTBE), solvent composition (MeOH: H_2_O: n-BuOH), solvent acidity and volume (1–9 mL), sample weight (5–40 mg) and filter types (Nylon, PVDF, PTFE, FG).

The optimized method for the extraction of lyophilized biomass is described as follows: 10 mg of lyophilized biomass was weighed in an Eppendorf micro-centrifuge tube (2.0 mL) and 1.5 mL of extraction solvent (75% MeOH: 25% H_2_O) was added. The mixture was shaken thoroughly with vortex and afterwards it was sonicated for 15 min in a sonication bath (Bandelin Sonorex Super RK106), in order to induce cell lysis. The mixture was then centrifuged (Sanyo Harrier 18/80 Refrigerated MSE) at 4000 rpm for 10 min at room temperature and the supernatant was separated from the pellet. The extraction was repeated one more time with 1.5 mL 75% MeOH :25% H_2_O and a third time with 1.5 mL n-BuOH. All the supernatants were pooled together and filtered using PVDF 0.45 μm Whatman filters. 2.5 mL of the filtrate was transferred in a test tube and was evaporated to dryness under a gentle nitrogen stream. The residue was re-dissolved with 1000 μL MeOH: H_2_O (5:95 v/v) and sonicated for 5 min. The final solution was transferred to an autosampler glass vial and analyzed by LC-MS/MS.

#### Method B

Filters with biomass were analyzed for CYN, ANA-a, NOD and 12 MCs (dmMC-RR, MC-RR, MC-YR, MC-HtyR, dm^3^MC-LR, MC-LR, MC-HilR, MC-WR, MC-LA, MC-LY, MC-LW, MC-LF), STX and neoSTX using a modified version of ISO 20179:2005^110^. More specifically, the filters were lyophilized and weighed. Subsequently they were placed in eppendorf tubes with 9 mL of an extraction mixture containing 75% MeOH: 25% H_2_O. They were sonicated for 15 min and centrifuged at 4000 rpm for 10 min (20 °C). The supernatant was filtered through a Whatman PVDF 0.45 μm filter. 3 mL of the filtered supernatant was transferred in a glass test tube and was evaporated to dryness under a gentle nitrogen stream. The residue was re-dissolved with 500 μL MeOH: H_2_O (5:95 v/v), sonicated for 5 min and analyzed by LC-MS/MS.

#### Methods C and D

Filtered water samples were analyzed for CYN, ANA-a, NOD and 12 MCs (dmMC-RR, MC-RR, MC-YR, MC-HtyR, dm^3^MC-LR, MC-LR, MC-HilR, MC-WR, MC-LA, MC-LY, MC-LW, MC-LF) using the dual cartridge Solid Phase Extraction (SPE) process^74^. For STX and neoSTX direct analysis was carried out, without any SPE pretreatment.

### LC-MS/MS analysis

A Finnigan Surveyor LC system, equipped with a Surveyor AS autosampler (Thermo, USA), coupled to a TSQ Quantum Discovery Max triple-stage quadrupole mass spectrometer (Thermo, USA), with electrospray ionization (ESI) source was employed for the chromatographic separation and detection of target analytes. Xcalibur software 2.0 was used to control the MS parameters, for data acquisition and data analysis.

LC-MS/MS analysis of CYN, ANA-a, and 12 MCs (dmMC-RR, MC-RR, MC-YR, MC-HtyR, dm^3^MC-LR, MC-LR, MC-HilR, MC-WR, MC-LA, MC-LY, MC-LW, MC-LF) was performed as it was previously described by a previous study^74^. Briefly, chromatographic separation was performed with reversed phase liquid chromatography (RPLC) column Atlantis T3 (2.1 mm × 100 mm, 3 μm) from Waters (Ireland). A gradient elution program was applied with solvents (A) ACN and (B) water, both containing 0.5% FA. The gradient started at 5% A (held for 3 min), which increased to 20% A in 1 min (held for 2 min), further to 35% A in 1 min (held for 7 min), 70% A in 14 min and finally 90% in 1 min (held for 3 min). An equilibration time of 10 min was kept after each sample run. Flow rate was set at 0.2 mL min^−1^ with 20 μL injection volume and column temperature was set at 30 °C.

STX and neo-STX were separated using a SeQuant ZIC-Hydrophilic Interaction Chromatography (HILIC) 150 mm × 2.1 mm, 3.5 mm column (Merck). A gradient elution program with water, ammonium acetate, acetonitrile and formic acid as solvents was used^76^. Selected molecular and fragment ion transitions, were according to Dell’Aversano *et al*.^111^.

Electrospray Ionization (ESI) in positive mode was used for ionization of all analytes. Multiple Reaction monitoring (MRM) mode was applied for the detection of CTs, using the three most intense and characteristic precursor/product ion transitions for each analyte. Identification of target CTs was based on three criteria: (1) retention time (t_R_) of compounds (2) three characteristic precursor/product ion transitions and (3) two calculated ratios of precursor/product ion transitions, achieving 5.5 “identification points” (IPs), in compliance with the strictest confirmation criteria of European Directives^112^. An example of the obtained MRM chromatograms is given in Fig. S8 for the sample from Lake Doirani, along with ratios of ion transitions in comparison to the corresponding ratios obtained by a standard solution (100 μg L^−1^).

For the calculation of TMCs, mean and median values, only the detected CT concentrations were taken into account, including the estimated values between LOD and LOQ.

### Matrix effects of extracted biomass

In order to assess the matrix effects of biomass extract on the quantitative determination of selected CTs, a series of experiments were performed. For biomass analysis, a sample which did not contain CTs was extracted according to the method previously described (method A), and an appropriate amount of analyte mixture containing CYN, ANA-a, MC-RR, MC-LR, STX and neoSTX was spiked in the final reconstituted solvent, in order to obtain a nominal concentration of 100 μg L^−1^. The spiked samples were analyzed using the above mentioned LC-MS/MS methods and they were compared to standard mixture solutions of the compounds at the same concentration level.

### Validation of methods

The methods were validated in order to assess specificity, linearity, precision (repeatability and reproducibility), accuracy (% recovery) and limits of detection (LODs)/quantification (LOQs). Blank samples were analyzed to assess method specificity. The linearity of each method was evaluated by analyzing in triplicates standard solutions at eight different concentrations (1, 2, 5, 10, 20, 50, 100, 250 μg L^−1^) for all CTs.

Limits of detection (LODs), accuracy and precision of methods C and D, used for the determination of CTs in water, are thoroughly described in past studies^74,76^. In the case of biomass/filter analysis (methods A and B, respectively), LOD calculation (expressed in ng mg^−1^ dw) was based on the LOD of each analyte in the extract (μg L^−1^), taking into consideration that 10 mg of biomass were extracted. Trueness and precision were evaluated by analyzing a toxins-free lyophilized biomass sample, spiked with a mixture of 12 MCs, CYN, ANA-a, STX and neoSTX at two content levels (3 and 30 ng mg^−1^), in six replicates at three different working days (n = 18).

### Risk assessment

In order to evaluate the possible human health hazards related to the presence of CTs in lake water, a risk-assessment approach has been carried out, taking into consideration the possible exposure routes and available guidelines. The TMCs found in the samples of the present study were calculated and compared to the guidance values and thresholds, established by WHO^41^. Finally, the amount of surface water that has to be accidentally consumed during swimming, in order to reach the threshold tolerable daily intake (TDI) set by WHO^43^, was also calculated for an average adult and child, based on the following formula:

1 $$V=\frac{T\,x\,bw}{C}$$

where:

V: amount of water (L) that have to be involuntarily ingested in order to reach each threshold.

T: threshold (TDI) value (μg kg^−1^ body weight).

bw (body weight): assuming child (10 kg) or adult (60 kg) average body weight (kg).

C: TMCs concentration: sum of intracellular and extracellular (μg L^−1^).

Assuming that each adult or child accidentally consumes 200 mL of water each day they swim^41^, the amount of orally ingested toxins and the percentage of TDI reached for a day’s swim, were also calculated.

## Electronic supplementary material

Supplementary Information

## Data Availability

All data generated or analyzed during this study are included in this published article (and the Supplementary Information files).
