# Supplementary material for: Occurrence and diversity of cyanotoxins in Greek lakes
Source: Sci Rep. 2018 Dec 14;8:17877. doi: 10.1038/s41598-018-35428-x (PMC6294760; doi:10.1038/s41598-018-35428-x)
Supplement: Supplementary file 1 — Supplementary Information [file 41598_2018_35428_MOESM1_ESM.docx]

**SUPPLEMENTARY INFORMATION**

**Occurrence and diversity of cyanotoxins in Greek lakes**

Christophoros Christophoridis^a^, Sevasti-Kiriaki Zervou^a^, Korina Manolidi^a^, Matina Katsiapi^b^, Maria Moustaka-Gouni^b^, Triantafyllos Kaloudis^a, c^, Theodoros M. Triantis^a^, Anastasia Hiskia*^a^

^a^ Institute of Nanoscience and Nanotechnology, National Center for Scientific Research “Demokritos”, Patr. Grigoriou E’ & Neapoleos 27, 15341, Athens, Greece.

^b^ School of Biology, Aristotle University of Thessaloniki, 54124 Thessaloniki, Greece

^c^ Water Quality Control Department, Athens Water Supply and Sewerage Company - EYDAP SA, Athens, Greece

* Corresponding Author. Tel.: +302106503643; Fax: +302106511766. E-mail address: a.hiskia@inn.demokritos.gr

**TABLES**

**Table S1.** Cyanotoxin occurrence in Greek Lakes – past studies

| **Lakes and reservoirs** | **Sample type** | **Time Period** | **Dominant cyanobacteria species** | **Occurrence of cyanotoxins** | **Analytical method** | **Reference** |
| --- | --- | --- | --- | --- | --- | --- |
| Lake Kastoria | Filtered Water | 2014 |  | [D-Asp^3^]MC-RR 1.7 μg L^-1^  MC-RR 63 μg L^-1^  MC-YR 3.6 μg L^-1^ | SPE-LC-MS/MS | [^1^](#_ENREF_1) |
| Lake Marathonas |  | 2015 |  | [D-Asp^3^]MC-LR 0.48 μg L^-1^  MC-LR 9 μg L^-1^  MC-HilR 0.42 μg L^-1^  MC-WR 0.51 μg L^-1^  MC-LA 0.54 μg L^-1^  MC-LY 0.16 μg L^-1^ |  |  |
| Lake VIstonis | Filtered biomass | July - August 2014 | *A. favaloroi, Pseudanabaena limnetica, Limnothrix sp., Merismopedia punctata, unidentified dinophyte, Scenedesmus spp., Monoraphidium griffithii, Nitzschia acicularis* | STXs (STX and neo-STX) were detected in the lake seston (42 and 17 ng mg^-1^ phytoplankton dw) | LC-MS/MS | [^2^](#_ENREF_2) |
| 36 lakes and reservoirs of Greece | Biomass | 1996-2004 | *Microcystis aeruginosa*  *Microcystis aeruginosa*  *Microcystis spp.*  *Microcystis aeruginosa*  *Anabaena flos-aquae*  *Jaaginema subtilissimum*  *Aphanizomenon flos-aquae* | Dominant MC-RR, MC-LR, MC-YR  MCs found in 95% of samples  Intracellular MC exceeding 10000 μg L^-1^ | ELISA, PPIA,  LC-DAD | [^3^](#_ENREF_3) |
| Lake Doirani | Biomass | 2009-2011 | *Microcystis wesenbergii* | Intracellular toxin concentration  MCs (3.9 – 108 μg L^-1^) STX (0.4 – 1.2 μg L^-1^)  STX (0.4 – 1.2 μg L^-1^)  CYN (0.3 -2.8 μg L^-1^) | ELISA  for MCs, CYN and STXs | [^4^](#_ENREF_4) |
| Kerkini Reservoir |  |  | *C. raciborskii* |  |  |  |
| Lake Volvi |  |  | *Anabaena perturbata, C. raciborskii* |  |  |  |
| Lake Kastoria |  |  | *Microcystis wesenbergii* |  |  |  |
| Lake Pamvotis |  |  | *Microcystis Novacekii, Microcystis viridis* |  |  |  |
| Lake Karla |  |  | *Limnothrix redekei, Planktothrix*  *agardhii, Anabaenopsis elenkinii* |  |  |  |
| Lake Pamvotis | Water and biomass | 1/2008 – 2/2009 | *Aphanocapsa sp., Merismopedia sp., Microcystis wesenbergii, Microcystis flos-aquae, Microcystis aeruginosa, Microcystis cf. Panniformis, Limnothrix redekei, Planktolyngbya circumcreta, Planktolyngbya limnetica, Anabaena flos-aquae, Anabaena spiroides, Anabaena crassa, Anabaena viguieri, Anabaenopsis elenkinii, Aphanizomenon issatschenkoi, Aphanizomenon flos-aquae* | Cell-bound MCs: <1 μg L^-1^- 19 μg L^-1^ eq.  extracellular MCs: <1 μg L^-1^- 9 μg L^-1^ eq.  STX in two samples 1.3 μg L^-1^ and 2.1 μg L^-1^ eq.  No CYN was detected | ELISA  for MCs, CYN and STXs | [^5^](#_ENREF_5) |
| Lake Karla | Biomass and water | 5/2011 | \| *Anabaenopsis nadsonii, Mastigocladopsis sp., Limnothrix sp., Synechococcus sp.* \| \| --- \| | Cell-bound MCs: 4.19 – 5.50 μg L^-1^  extracellular MCs:2.03 –3.01 μg L^-1^ | ELISA  for MCs | [^6^](#_ENREF_6) |
| Lake Marathonas | Water and biomass |  | *-* | MCs in water:  MC-RR: 2 - 174 ng.L^-1^  MC-LR: 2 – 451 ng.L^-1^  MC-YR: 2 - 717 ng.L^-1^  MC-LA: 5 – 8 ng L^-1^  MCs in biomass:  MC-RR: 1956 μg g^-1^  MC-LR: 382 μg g^-1^  MC-YR: 555 μg g^-1^ | SPE, LC-ESI-MS/MS | [^7^](#_ENREF_7) |
| Lake Karla | Water  Biomass  Fish tissue | 2010 | *Anabaenopsis elenkinii, Sphaerospermopsis, Planktothrix agardhii*  *Other cyanobacteria:*  *Aphanocapsa delicatissima, Lemmermaniella pallida, Limnothrix redekei, Merismopedia tenuissima, Merismopedia warmingiana, Microcystis aeruginosa, Microcystis panniformis, Planktolyngbya cf. limnetica, Pseudanabaena limnetica,*  *Raphidiopsis mediterranea, Snowella litoralis, Sphaerospermopsis aphanizomenoides* | extracellular MCs: 0.75 - 3.90 μg L^-1^  Cell-bound MCs in seston: 1.01 - 9.83 μg L^-1^  Fish tissue C. carpio liver : 181.91 ng g^-1^  Detected also in kidney>brain>intestine tissues of Cyprinus carpio fishes | ELISA  for MCs | [^8^](#_ENREF_8) |
| Lake Karla | Biomass | 3-4/2010 | *Plastids, Oscillatoriales, Chroococcales (Planktothrix agardhii, Planktothrix rubescens, Anabaena sp., and Anabaenopsis*  *Elenkinii)* |  |  | [^9^](#_ENREF_9) |
| Lake Marathonas | biomass | 2007, 2008 | *Cyanobacteria phylotypes -> Chroococcales* |  |  | [^10^](#_ENREF_10) |
| Lake Pamvotis | Water and Biomass | January – December 2008 | *Microcystis sp.*  *Anabaena (June and July)* | Extracellular MCs:0.0121 – 7.88 μg MC-LR eq. L^-1^  Intracellular MCs: 0.15 – 15.21 μg MC-LR eq. L^-1^  Maximum values during July - October | ELISA for MCs  HPLC-UV for confirmation of MC-LR presence | [^11^](#_ENREF_11) |
| Lake Marathonas | biomass | 2007, 2008 | *Microcystis aeruginosa, Chroococcus disperses, Chroococcales sp.* | - | - | [^12^](#_ENREF_12) |
| 10 Greek lakes | Water |  |  | ANA-a:  Sample A: 9 ng L^−1^  Sample B: 5 ng L^−1^ | LC-MS/MS | [^13^](#_ENREF_13) |
| Lake Trichonis | Water, biomass and fish tissue | Spring 2005 | *Oscillatoria sp., Anabaena sp., Aphanizomenon flos-aquae* | **MCs** detected in water, scum and tissues of fish *Carassius gibelio* | ELISA  MC concentration values are given in Charts | [^14^](#_ENREF_14) |
| Lake Lyshimachia |  |  | *Oscillatoria sp., Anabaena sp.,*  *Aphanizomenon flos-aquae* | **MCs** detected in water, scum and tissues of fish *Carassius gibelio* |  |  |
| Lake Pamvotis |  |  | *Microcystis sp., Anabaena sp.* | **MCs** detected in water, scum and tissues of fish *Carassius gibelio* |  |  |
| Lake Mikri prespa |  |  | *Microcystis sp.* | **MCs** detected in water, scum and tissues of fish *Carassius gibelio* |  |  |
| Lake Vegoritis |  |  | *Aphanizomenon flos-aquae, Anabaena sp.* | **MCs** detected in water, scum and tissues of fish *Carassius gibelio* |  |  |
| Lake Kastoria |  |  | *Microcystis sp., Anabaena sp.* | **MCs** detected in water, scum and tissues of fish *Carassius gibelio* |  |  |
| Lake Zazari |  |  | *Microcystis sp.* | **MCs** detected in water, scum and tissues of fish *Carassius gibelio* |  |  |
| Lake Petron |  |  | *Microcystis sp., Aphanizomenon flos-aquae* | **MCs** detected in water, scum and tissues of fish *Carassius gibelio* |  |  |
| Lake Chimaditis |  |  | *Microcystis sp., Anabaena sp., Oscillatoria sp.* | **MCs** detected in water, scum and tissues of fish *Carassius gibelio* |  |  |
| Lake Koronia |  |  | *Microcystis sp., Anabaena sp.* | MCYST in **water:** 3748.6 ng L^-1^  MCYST in **scum:** 15,896 ng L^-1^  **MCs** detected also in tissues of fish *Carassius gibelio* |  |  |
| Lake Volvi |  |  | *Anabaena sp., Aphanizomenon flos-aquae, Juaginema subtilissimum, Limnothrix redekei, Planktolyngbya limnetica* | MCYST in **water:** 209.56 ng L^-1^  MCYST in **scum:** 1086 ng L^-1^  **MCs** detected also in tissues of fish *Carassius gibelio* |  |  |
| Lake Doirani |  |  | *Anabaena sp., Aphanizomenon flos-aquae, Juaginema subtilissimum, Limnothrix*  *redekei, Planktolyngbya limneticae* | **MCs** detected in water, scum and tissues of fish *Carassius gibelio* |  |  |
| Lake Kerkini |  |  | *Microcystis sp.* | **MCs** detected in water, scum and tissues of fish *Carassius gibelio* |  |  |
| Lake Mornos  Lake Yliki Lake Marathonas  Lake Kastoria | Water |  |  | Water samples from Lakes Mornos and Lake Yliki were negative for cyanotoxins  Water samples from lake Marathonas contained:  MC-RR 0.005-0.060 μg L^-1^  MC-LR 0.004 – 0.014 μg L^-1^  MC-YR 0.001 – 0.004 μg L^-1^  Water samples from lake Kastoria contained:  MC-RR 0.008 μg L^-1^  MC-LR 0.007 μg L^-1^  MC-YR <LOD μg L^-1^ | Elisa, PPIA, HPLC/PDA, LC-MS/MS | [^15^](#_ENREF_15) |
| Lake Koronia | Biomass | 9/2004 | *Anabaena aphanizomenoides, Anabaena sp., Anabaenopsis elenkinii, Merismopedia minima, Planktothrix sp., unidentified oscillatoriales* |  |  | [^16^](#_ENREF_16) |
| Lake Pamvotis | Biomass and fish tissues | 8/2004 – 12/2005 | *Microcystis sp. and*  *Anabaena sp. (and/or) Aphanizomenon sp.* | MCs in **water**: 0.01 μg L^-1^- 19.5 μg L^-1^  MCs in **fish tissues:**  bivalve 49.8 - 102.2 ng g^-1^ w/w  muscle: 2.06 - 4.71 ng g^-1^ w/w  liver 14.2 - 56.2 ng g^-1^ w/w | ELISA  (Abraxis Microcystins Kit) | [^17^](#_ENREF_17) |
| Lake Pamvotis | Water, biomass and fish tissues | 2005 | *Microcystis sp and Anabaena sp* | MCYST in **water**: 0.310 – 2.4 μg L^-1^  MCYST in **blooms**: 3.1 – 11.6 μg L^-1^  MCYST in **fish tissues of Carassius gibelio**:  Liver (mean: 275.1±84.5 ng g^-1^)  Intestine (mean: 233.51±196.5 ng g^-1^)  kidney (mean: 155.8±35.8 ng g^-1^)  brain (mean: 38.5±26.9 ng g^-1^)  gonads (mean: 21.02± 9.16 ng g^-1^)  muscle (mean: 16.05±11.97 ng g^-1^) | ELISA  (Abraxis Microcystins Kit) | [^18^](#_ENREF_18) |
| Lake Kastoria | Biomass samples | 11/1998 – 10/1999 | *4 Dominant cyanobacterial species:*  ***Limnothrix redekei, Microcystis aeruginosa, Cylindrospermopsis raciborskii and Aphanizomenon gracile***  *Other cyanobacteria (Aphanocapsa elachista, Aphanothece sp., Chroococcus limneticus, Merismopedia tenuissima, Microcystis flos-aquae, Microcystis ichthyoblabe, Microcystis novacekii, Microcystis wesenbergii, Pannus spumosus, Snowella lacustris, Synechococcus sp., Woronichinia naegeliana, Anabaena cf. aphanizomenoides, Anabaena flos-aquae, Anabaena viguieri, Aphanizomenon gracile, Aphanizomenon issatschenkoi)* |  |  | [^19^](#_ENREF_19) |
| Lake Kastoria | Biomass | 3/1999 – 10/1999 | *Cylindrospermopsis raciborskii, Aphanizomenon spp. And Microcystis aeruginosa* | **MCs**: <LOD - 34.0 μg MC-LR equivalents L^-1^ | Envirologix Microcystin  Plate Kit  & confirmed by HPLC | [^20^](#_ENREF_20) |
| Lake Yliki |  |  |  | **MCs** in Fish tissues- *Cyprinus carpio* | PPIA,  ELISA, & HPLC | [^21^](#_ENREF_21) |
| River Gallikos |  |  |  | **MCs** in Frog tissues - *Rana ridibunda* |  |  |
| Lake Kastoria |  |  |  | **MCs** in Mussel *- Anodonta* sp.  **MCs** in Fish tissues *- Carassius gibelio, Perca fluviatilis* |  |  |
| Lake Kerkini |  |  |  | **MCs** in Fish tissues *- Carassius gibelio,*  *Cyprinus carpio* |  |  |
| Lake Pamvotis |  |  |  | **MCs** in Fish tissues *- Cyprinus carpio,*  *Silurus aristotelis,*  *Acipenser gueldenstaedtii,*  *Carassius auratus,*  *Rutilus rubilio, Cyprinus caprio, Silurus glanis, Carassius auratus,*  **MCs** in frog tissues *- Rana eperotica,*  **MCs** in Water snail *- Viviparus contectus* |  |  |
| Lake Lysimachia | Filtered biomass | July 1999 | *Aphanizomenon ovalisporum, Pseudanabaena sp.,*  *Planktothrix mougeotii* | MC–LR in water - 0.9 μg L^-1^ | HPLC for MC–LR,  [D-Asp^3^]MC–LR, [Dha7]MC–LR,  [D-Asp^3^,Dha7]MC–LR, MC-RR, [D-Asp^3^]MC-RR, [Dha7]MC-RR,  [D-Asp^3^,Dha7] MC-RR, MC-LA, MC-YR | [^22^](#_ENREF_22) |
| Lake Trichonis | Filtered biomass | July 1999 | *Aphanizomenon ovalisporum,*  *Anabaena sp.* | Cyanotoxins were not detected |  |  |
| Lake Kastoria |  |  | *Limnothrix redekei*  *(3 strains: 007a, 165a, 165c)* |  |  | [^23^](#_ENREF_23) |
| Lake Vistonis | Filtered biomass | 1994, 1995, 1999 and 2000 (Warm period) | *Microcystis aeruginosa, Microcystis spp* | Total MCs 317.2 ng mg^-1^  (MC-LR ) | HPLC – DAD  Identification for  MC-LR, [D-Asp^3^] MC-LR, [Dha7] MC-LR, [D-Asp^3^, Dha7] MC-LR, MC-RR, [D-Asp^3^] MC-RR, [Dha7] MC-RR, [D-Asp^3^, Dha7] MC-RR, MC-LA, and MC-YR | [^24^](#_ENREF_24) |
| Kerkini Reservoir |  |  | *Anabaena spiroides, Microcystis aeruginosa, Microcystis flos-aquae, Microcystis wesenbergii, Microcystis spp* | Total MCs 43.9 – 598.6 ng mg^-1^  (MC-LR, MC-RR, [Dha7]MC-RR, MC-YR) |  |  |
| Lake Mikri Prespa |  |  | *Microcystis wesenbergii, Microcystis spp* | Total MCs 1153.3 ng mg^-1^  (MC-LR, [D-Asp^3^] or [Dha7]MC-LR, MC-RR, MC-LA) |  |  |
| Lake Zazari |  |  | *Microcystis aeruginosa, Microcystis spp* | Total MCs 50.3 ng mg^-1^  (MC-LR, MC-RR) |  |  |
| Lake Kastoria |  |  | *Cylindrospermopsis raciborskii, Limnothrix redekei, Microcystis aeruginosa, Microcystis flos-aquae, Microcystis novacekii* | Total MCs 114.5 – 2564.3 ng mg^-1^  (MC-LR, [D-Asp^3^] or [Dha7]MC-LR, MC-RR, [Dha7]MC-RR, MC-YR, MC-LA) |  |  |
| Lake Pamvotis |  |  | *Anabaena flos-aquae, Microcystis aeruginosa, Microcystis spp* | Total MCs 778.1 – 1132.0 ng mg^-1^  (MC-LR, [D-Asp^3^] or [Dha7]MC-LR, MC-RR) |  |  |
| Lake Amvrakia |  |  | *Anabaena perturbata, Anabaena viguieri, Microcystis spp* | Total MCs 42.2 – 166.9 ng mg^-1^  (MC-LR, MC-RR) |  |  |
| Kerkini Reservoir |  |  | *M. aeruginosa, M. flos-aquae, M. ichthyoblabe, M. novacekii, M. wesenbergii, Pseudanabaena sp., Anabaena flos-aquae, Anabaena viguieri, A. spiroides, A. issatschenkoi* | - | only toxin producing species and first occurring species are reported | [^23^](#_ENREF_23) |
| Lake Amvrakia |  |  | *Microcystis aeruginosa, M. flos-aquae, M. novacekii, M. wesenbergii, Anabaena perturbata, A. viguieri* | - |  |  |
| Lake Doirani |  |  | *Microcystis aeruginosa, Jaaginema subtilissimum, Anabaena aphanizomenoides, Anabaena flos-aquae, Aphanizomenon flos-aquae* | - |  |  |
| Lake Kastoria |  |  | *Microcystis aeruginosa, Anabaena affinis, Microcystis ichthyoblabe, Microcystis novacekii, Microcystis wesenbergii, Anabaena flos-aquae, Anabaena viguieri, Cylindrospermopsis raciborskii* | - |  |  |
| Lake Mikri Prespa |  |  | *Microcystis aeruginosa, Microcystis wesenbergii, Anabaena lemmermannii,* | - |  |  |
| Lake Pamvotis |  |  | *Microcystis aeruginosa, M. flos-aquae, M. novacekii, M. viridis, M. wesenbergii, Anabaena flos-aquae, Aphanizomenon flos-aquae* | - |  |  |
| Lake Vistonis |  |  | *Microcystis aeruginosa, M. ichthyoblabe, M. wesenbergii, Anabaena sp.* | - |  |  |
| Lake Volvi |  |  | *Microcystis aeruginosa, Anabaenopsis cunningtonii, Microcystis novacekii, Anabaena circinalis, Cylindrospermopsis raciborskii* | - |  |  |
| Lake Zazari |  |  | *Microcystis aeruginosa, Microcystis*  *flos-aquae, Microcystis ichthyoblabe, Microcystis novacekii, Microcystis viridis, Microcystis wesenbergii, Pseudanabaena sp., Anabaena flos-aquae, Anabaena viguieri, Anabaena spiroides, Aphanizomenon issatschenkoi, Cylindrospermopsis raciborskii* | - |  |  |
| Vistonis |  |  | *Microcystis, Oscillatoria, Anabaena, Anabaenopsis* | Total MCs = 317.2 μg g^-1^  LD50 in mice = 1130 – 1500 mg kg^-1^ |  | [^25-27^](#_ENREF_25) |
| Volvi |  |  | *Aphanocapsa, Microcystis, Anabaena, Anabaenopsis, Aphanizomenon, Cylindrospermopsis* | LD50 in mice = 1500 mg kg^-1^ |  |  |
| Koronia |  |  | *Anabaenopsis* | LD50 in mice = 600 mg kg^-1^ |  |  |
| Kerkini |  |  | *Microcystis, Anabaena, Aphanizomenon* | Total MCs = 68.7 ± 24.8 μg g^-1^ |  |  |
| Doirani |  |  | *Microcystis, Anabaena, Anabaenopsis, Aphanizomenon* | - |  |  |
| Agras |  |  | *Aphanocapsa, Anabaena,* | - |  |  |
| Vegoritis |  |  | *Anabaena, Aphanizomenon* | - |  |  |
| Petron |  |  | *Microcystis, Aphanizomenon* | - |  |  |
| Mikri Prespa |  |  | *Aphanocapsa, Microcystis, Oscillatoria, Anabaena* | Total MCs = 1091 μg g^-1^ |  |  |
| Zazari |  |  | *Microcystis, Anabaena, Aphanizomenon, Cylindrospermopsis* | Total MCs = 50.3 μg g^-1^ |  |  |
| Cheimaditis |  |  | *Microcystis, Oscillatoria, Anabaena* | - |  |  |
| Kastoria |  |  | *Microcystis, Anabaena, Cylindrospermopsis* | Total MCs = 1638 ± 464 μg g^-1^  LD50 in mice = 40 – 1500 mg kg^-1^ |  |  |
| Asomaton |  |  | *Oscillatoria, Aphanizomenon, Cylindrospermopsis* | - |  |  |
| Polyphyton |  |  | *Microcystis, Anabaena, Aphanizomenon, Cylindrospermopsis* | - |  |  |
| Pamvotis |  |  | *Aphanocapsa, Microcystis, Oscillatoria, Anabaena, Aphanizomenon* | Total MCs = 958 ± 75 μg g^-1^ |  |  |
| Tavropos |  |  | *Oscillatoria, Anabaena* | - |  |  |
| Louros |  |  | *Microcystis, Oscillatoria, Anabaena* | - |  |  |
| Pournariou |  |  | *Other genera* | - |  |  |
| Kremaston |  |  | *Microcystis* | - |  |  |
| Saltini |  |  | *Other genera* | - |  |  |
| Voulkaria |  |  | *Microcystis, Oscillatoria, Aphanizomenon* | - |  |  |
| Amvrakia |  |  | *Microcystis, Anabaena* | Total MCs = 84 ± 41 μg g^-1^ |  |  |
| Kastrakiou |  |  | *Anabaena* | - |  |  |
| Ozeros |  |  | *Oscillatoria, Anabaena, Aphanizomenon* | - |  |  |
| Lysimachia |  |  | *Oscillatoria, Anabaena, Aphanizomenon* | - |  |  |
| Trichonis |  |  | *Oscillatoria, Anabaena, Aphanizomenon* | - |  |  |
| Mornos |  |  | *Anabaena* | - |  |  |
| Yliki |  |  | *Aphanocapsa, Oscillatoria, Anabaena, Anabaenopsis, Aphanizomenon, Cylindrospermopsis* | - |  |  |
| Paralimni |  |  | *Anabaena* | - |  |  |
| Marathonas |  |  | *Microcystis, Cylindrospermopsis* | - |  |  |
| Stymfalia |  |  | *Anabaena, Aphanizomenon* | - |  |  |
| Pinios |  |  | *Microcystis, Anabaena, Cylindrospermopsis* | - |  |  |
| Floka |  |  | *Microcystis, Oscillatoria, Anabaena* | - |  |  |
| Lake Porto Lagos (Vistonis) |  |  | *Anabaenopsis milleri Woronichin* | Probably MC-LR | TLC, HPTLC & HPLC-UV | [^28^](#_ENREF_28) |
| Lake Koronia |  |  | *Anabaenopsis milleri* | LD 50 = 600 mg kg^-1^ dw | Mice bioassay – LD 50 intraperitoneal injection | [^29^](#_ENREF_29) |
| Lake Volvi |  |  | *-* | LD 50 = 1500 mg kg^-1^ dw |  |  |
| Lake Kastoria |  |  | *Microcystis aeruginosa, Anabaena viguieri* | LD 50 = 40 - 1500 mg kg^-1^ dw |  |  |
| Lake Visthonis (or Porto Lagos) |  |  | *Anabaenopsis millen, Microcystis aeruginosa, Oscillatoria sp.* | LD 50 = 1130 - 1500 mg kg^-1^ dw |  |  |

**Table S2.** % Recovery of cyanotoxins in cyanobacterial biomass (method A)

|  | **Content Level 3 ng mg^-1^ dw** | | **Content Level 30 ng mg^-1^ dw** | |
| --- | --- | --- | --- | --- |
| **Cyanotoxin** | **Average** | **%RSD_R_** | **Average** | **%RSD_R_** |
| CYN | 33.1 | 5.8 | 35.4 | 7.6 |
| ANA-a | 60.4 | 6.6 | 62.9 | 6.3 |
| dmMC-RR | 97.0 | 8.1 | 105.0 | 8.5 |
| MC-RR | 90.9 | 11.4 | 105.2 | 6.9 |
| MC-YR | 113.2 | 23.5 | 114.6 | 12.0 |
| MC-HtyR | 104.4 | 14.4 | 117.6 | 6.2 |
| dm^3^MC-LR | 150.9 | 26.4 | 98.9 | 30.2 |
| MC-LR | 104.0 | 10.5 | 116.2 | 8.8 |
| MC-HilR | 134.5 | 14.0 | 102.6 | 24.2 |
| MC-WR | 91.0 | 24.4 | 105.5 | 11.7 |
| MC-LA | 133.9 | 10.3 | 109.4 | 28.5 |
| MC-LY | 120.2 | 13.8 | 118.9 | 17.0 |
| MC-LW | 154.5 | 11.0 | 110.8 | 20.1 |
| MC-LF | 130.1 | 8.1 | 129.2 | 13.3 |
| neoSTX | 33.0 | 12.2 | 35.1 | 10.2 |
| STX | 30.0 | 11.6 | 32.1 | 9.9 |

**Table S3.** LODs and LOQs of cyanotoxins in cyanobacterial biomass (method A)

| **Cyanotoxin** | **LOD**  **(ng mg^-1^ dw)** | **LOQ**  **(ng mg^-1^ dw)** |
| --- | --- | --- |
| CYN | 0.1 | 0.4 |
| ANA-a | 0.1 | 0.4 |
| dmMC-RR | 0.3 | 0.9 |
| MC-RR | 0.1 | 0.4 |
| NOD | 0.3 | 0.8 |
| MC-YR | 0.7 | 2.0 |
| MC-HtyR | 1.0 | 2.9 |
| dm^3^MC-LR | 0.5 | 1.6 |
| MC-LR | 0.6 | 1.9 |
| MC-HilR | 0.9 | 2.8 |
| MC-WR | 0.8 | 2.5 |
| MC-LA | 0.4 | 1.3 |
| MC-LY | 0.9 | 2.7 |
| MC-LW | 0.7 | 2.0 |
| MC-LF | 0.8 | 2.4 |

**Table S4.** Observed % matrix suppression of selected cyanotoxins in cyanobacterial biomass extract

|  | **Conc. Level 100 μg L^-1^** | |
| --- | --- | --- |
| **Cyanotoxin** | **Mean** | **%RSD_R_** |
| CYN | 68.0 | 7.8 |
| ANA-a | 29.5 | 5.7 |
| MC-RR | 7.8 | 3.3 |
| MC-LR | 11.2 | 2.3 |
| neoSTX | 27.1 | 7.3 |
| STX | 18.7 | 5.7 |

**Table S5.** Guideline levels for recreational water and tolerable daily intakes (TDI) set by WHO and OPHD for MCs, CYN and ANA-a.

| *Guidelines of WHO for safe practice in managing recreational waters* [*^30^*](#_ENREF_30) | | | |
| --- | --- | --- | --- |
| **Risk level** | **Cyanobacterial count (cells mL^-1^)** | **TMC concentration (µg L^-1^)** | |
| low risk | >2000 | < 10 | |
| moderate risk | 20000 | 10-20 | |
| high risk | > 100000 | > 20 | |
| *Guidelines set by the Oregon Public Health Division (OPHD)* [*^31^*](#_ENREF_31) *for recreational water* | | | |
| **Cyanotoxin** | **Total concentration (µg L^-1^)** | | |
| MCs | 10 | | |
| ANA-a | 20 | | |
| CYN | 6 | | |
|  | | | |
| *TDI values calculated by WHO* [*^32^*](#_ENREF_32) | | | |
| **Cyanotoxin** | **TDI - tolerable daily intake (µg kg^-1^ day^-1^)** | **NOAEL* (µg kg^-1^ day^-1^)** | **LOAEL** (µg kg^-1^ day^-1^)** |
| MCs | 0.04 | 40.0 | 100 |
| *TDI values calculated by the (OPHD)* [*^31^*](#_ENREF_31) | | | |
| **Cyanotoxin** | **TDI - tolerable daily intake (µg kg^-1^ day^-1^)** | | |
| MCs | 0.05 | | |
| ANA-a | 0.1 | | |
| CYN | 0.03 | | |

*NOAEL: No-observed-adverse-effect level

** LOAEL: lowest-observed-adverse-effect level

**Figures**

**
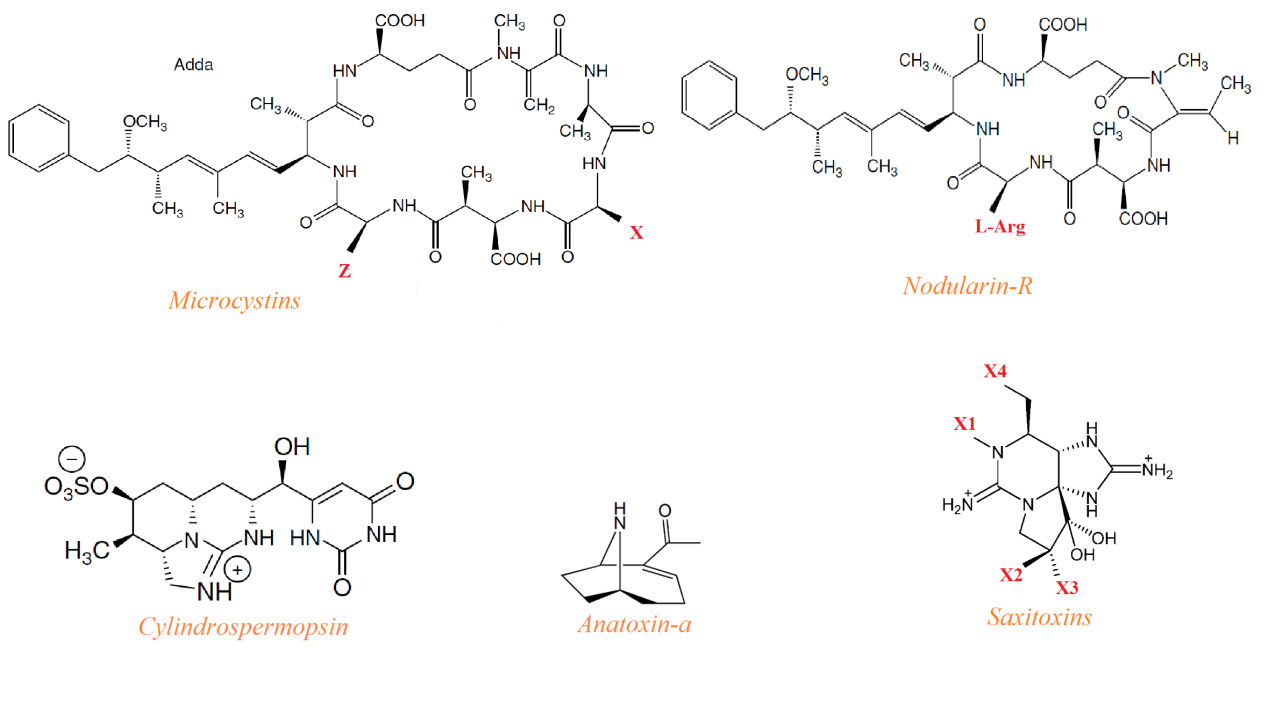
**

**Figure S1.** Chemical structures of main CT groups. For Microcystins, X and Z represent variable L-amino acids. For Saxitoxin, X_1_, X_2_, X_3_ = H and X_4_ = OCONH_2_. For neo-Saxitoxin, X1=OH, X_2_, X_3_ = H and X_4_ = OCONH_2_.


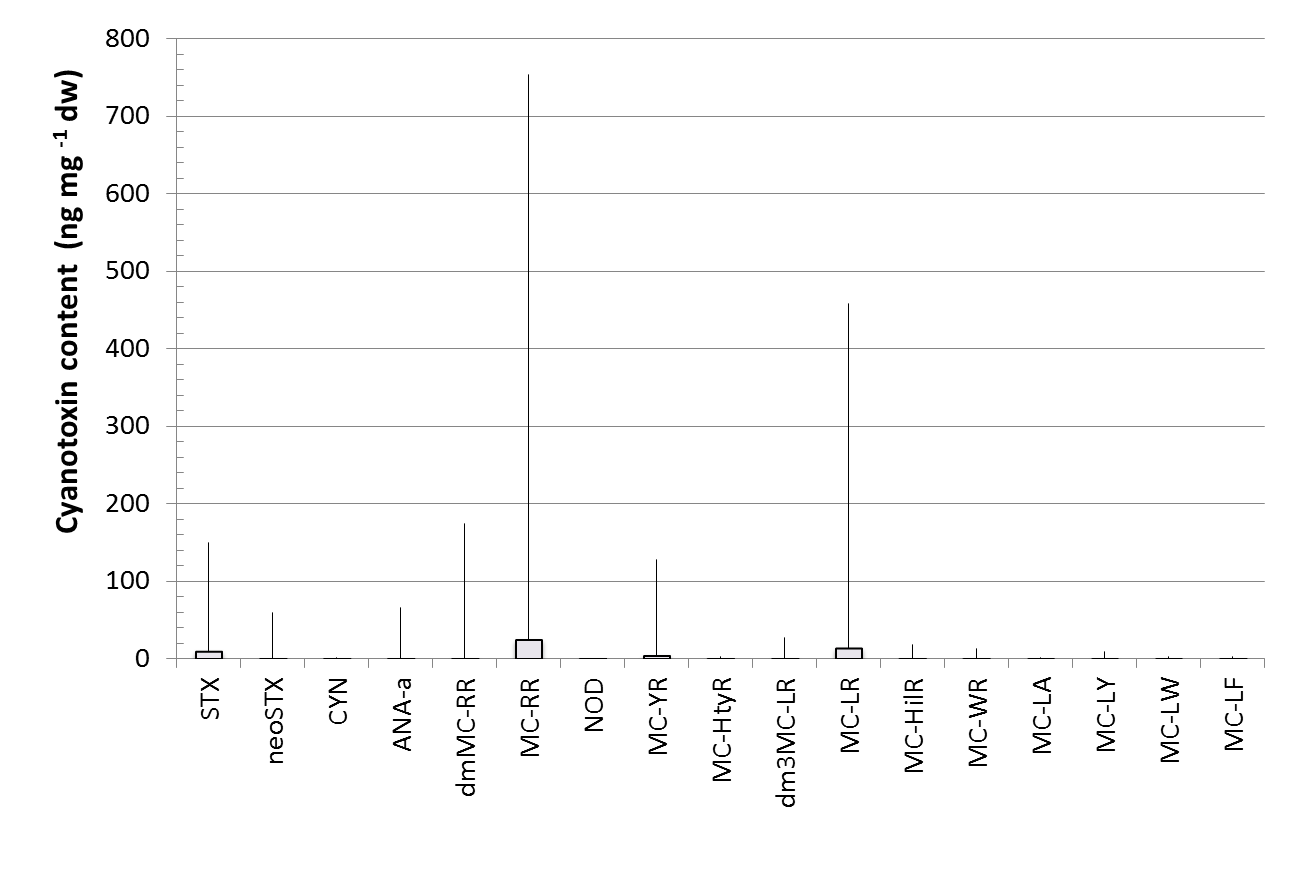


**Figure S2**. Content range of cyanotoxins in intracellular cyanobacteria biomass of Greek lakes, including the 25^th^ – 75^th^ percentile of determined values


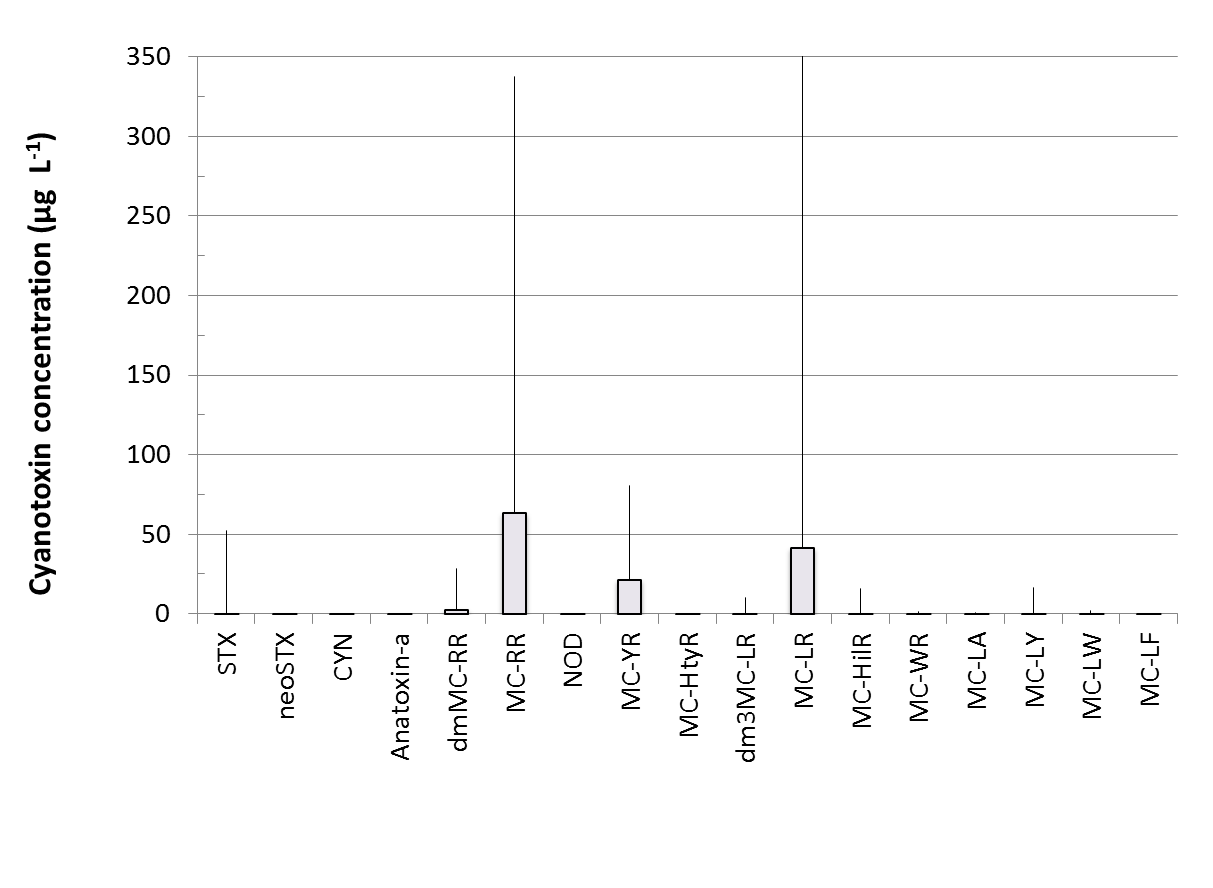


**Figure S3**. Concentration range of extracellular cyanotoxins detected in water samples of Greek lakes, including the 25^th^ – 75^th^ percentile of determined values


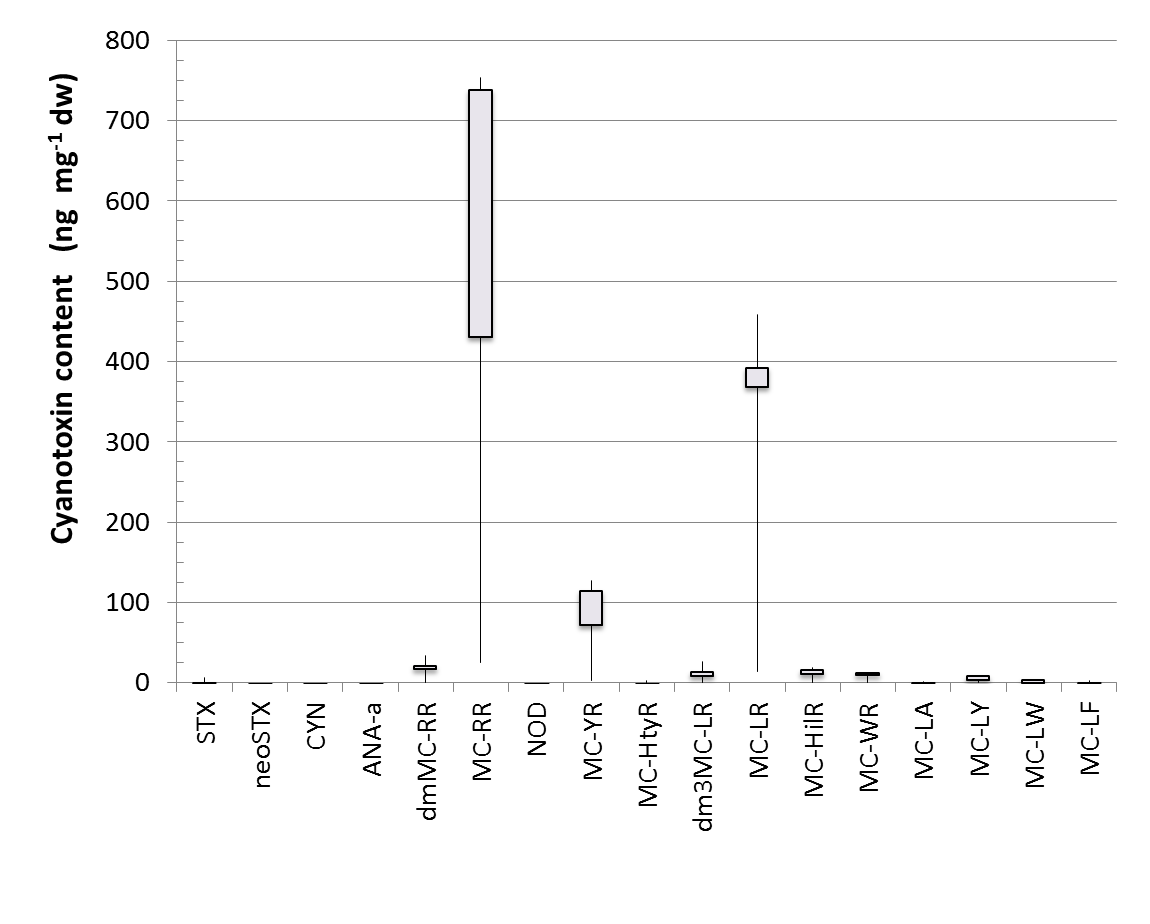


**Figure S4.** Content range of intracellular cyanotoxins detected in biomass obtained from lake Kastoria, with 25^th^-75^th^ percentile of determined values


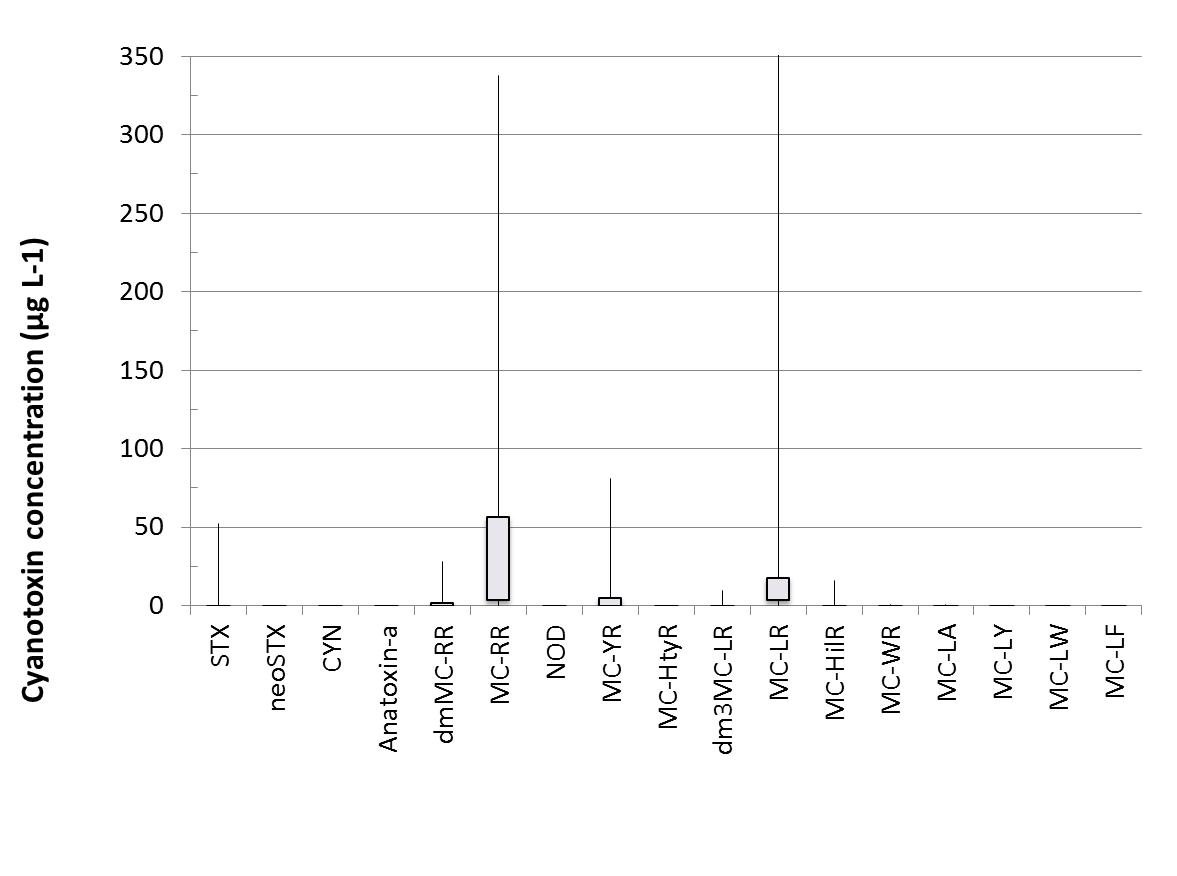


**Figure S5.** Concentration range of extracellular cyanotoxins detected in water obtained from lake Kastoria, with 25^th^-75^th^ percentile of determined values


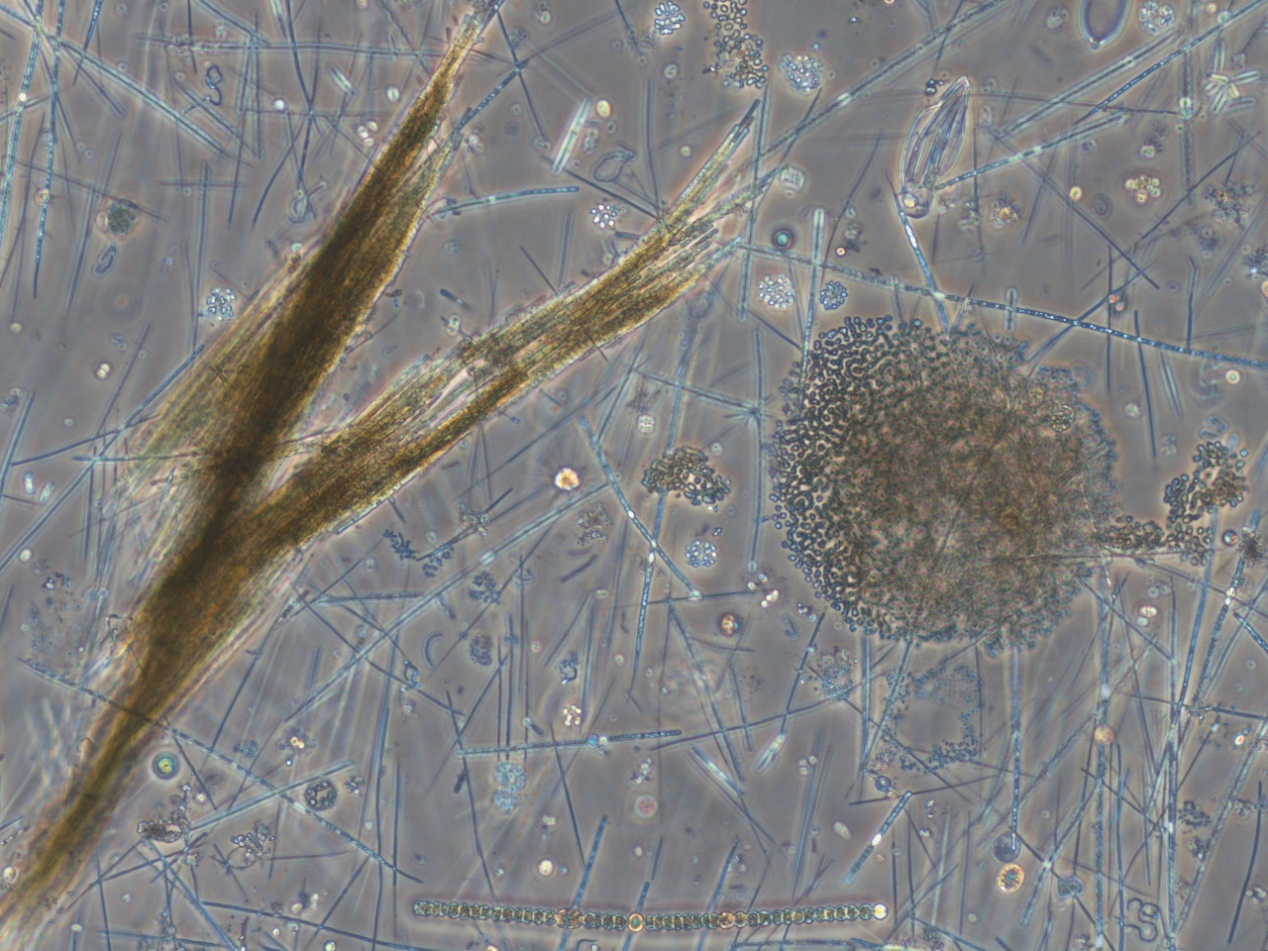


**Figure S6:** Micrograph of water sample from Lake Kerkini (07/06/2008). Conspicuous *Aphanizomenon flos-aquae* bundles, *Microcystis* colony and several other species of Nostocales, Oscillatoriales and Chroococcales.


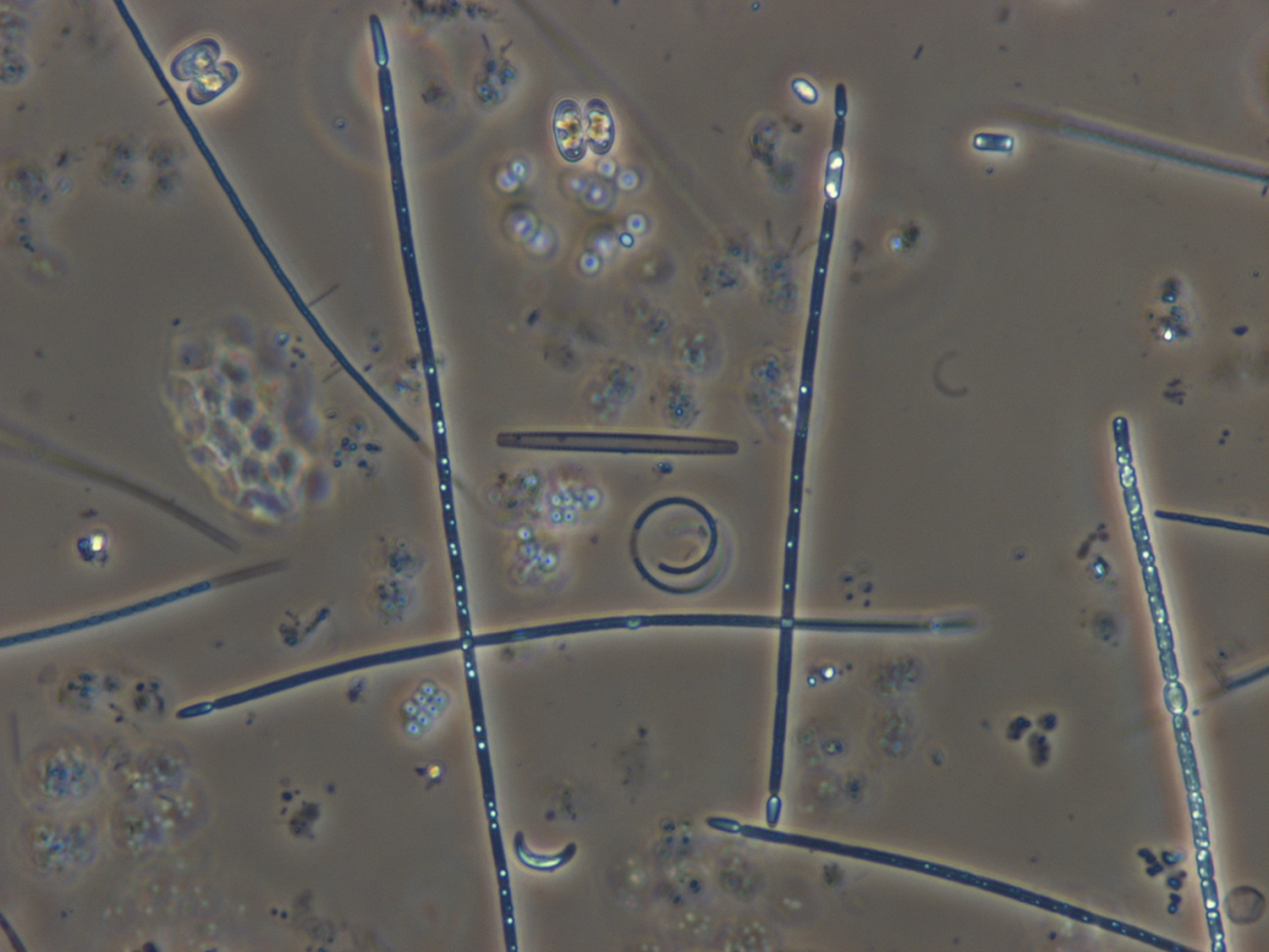


**Figure S7:** Micrograph of water sample from Lake Petron. Dominant cyanobacteria: *Cylindrospermopsis, Planktolyngbya, Aphanizomenon* and *Cyanodictyon*  species.


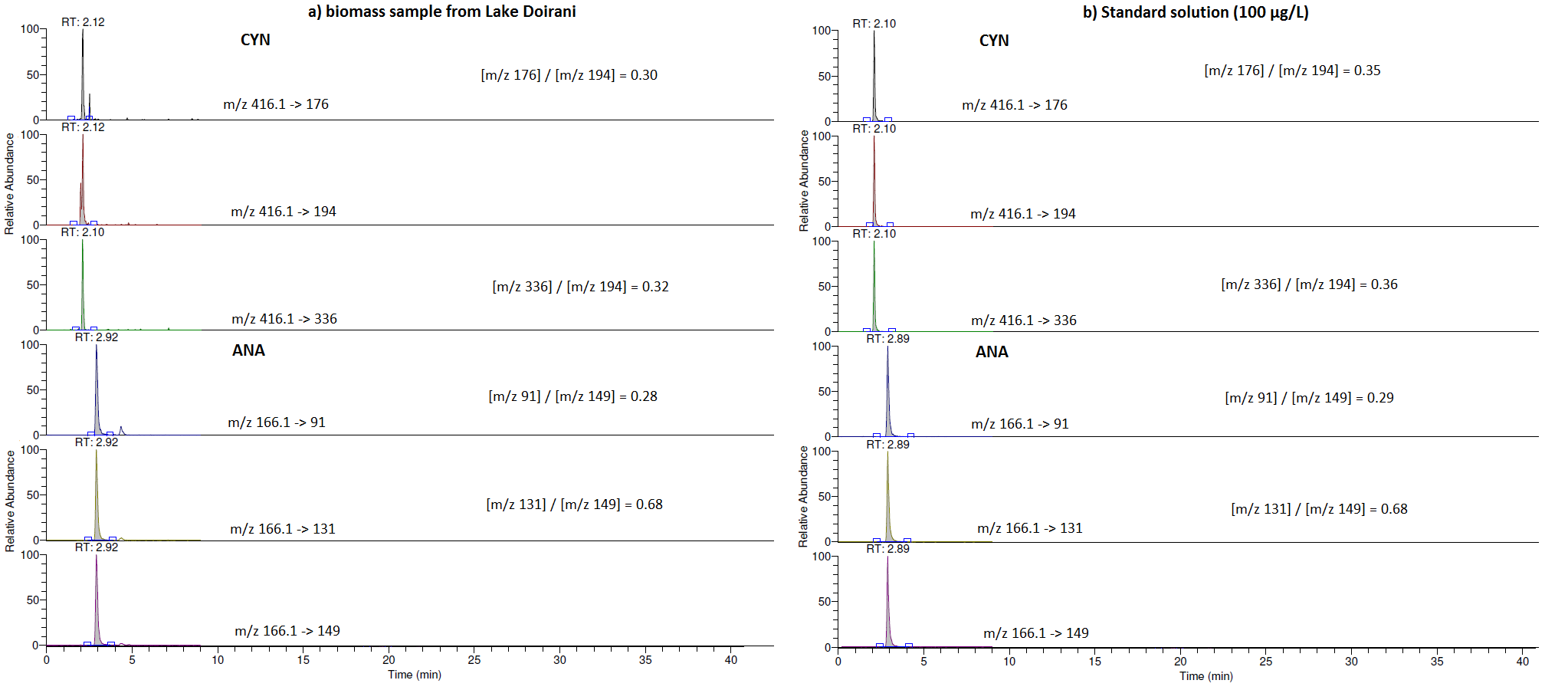


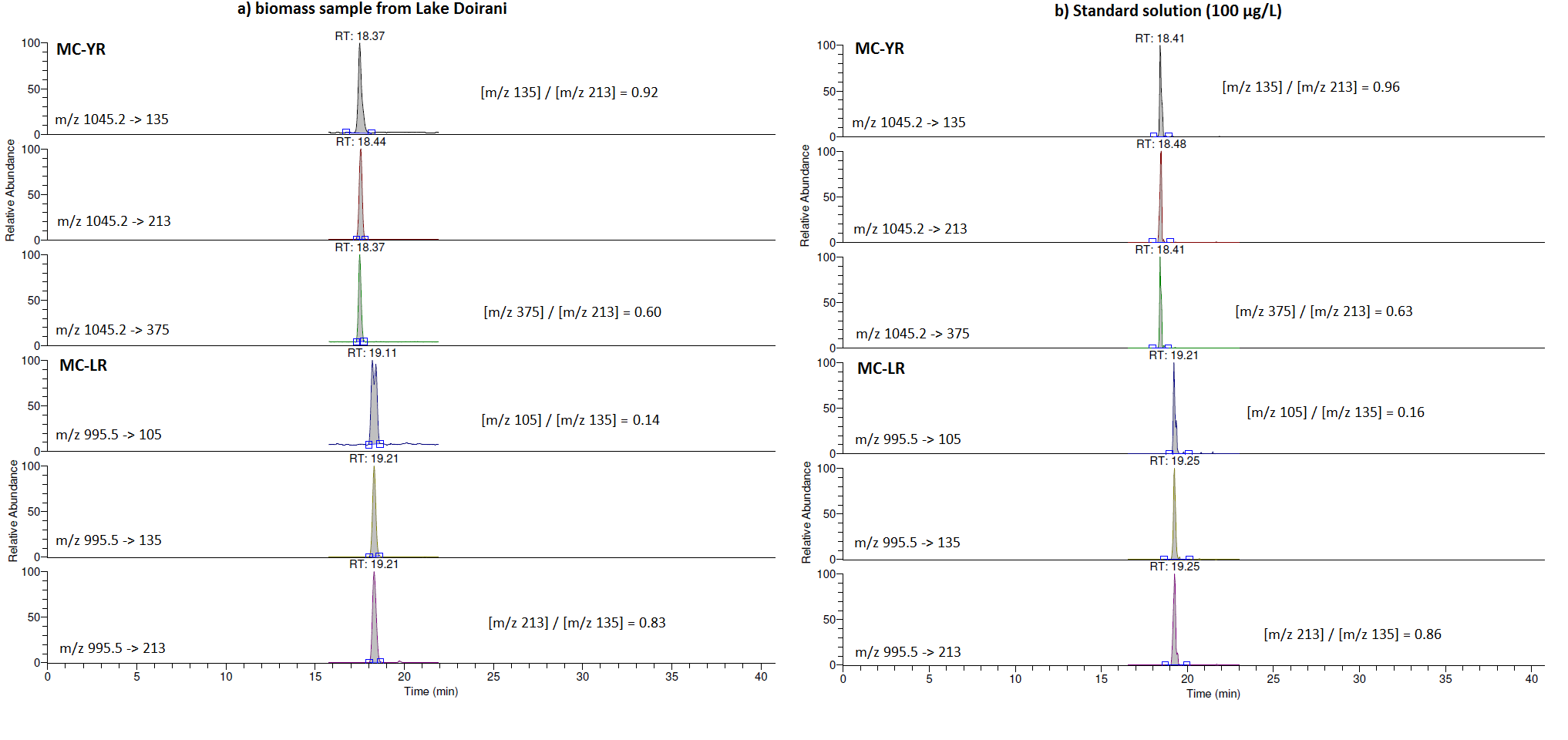


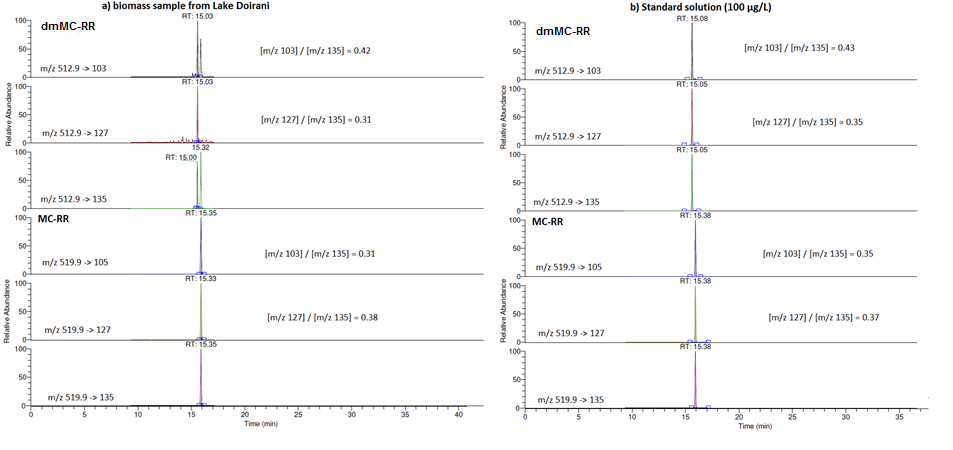


Figure S8. MRM chromatograms of identified cyanotoxins in the sample from Lake Doirani, along with ratios of ion transitions in comparison to a standard (100 μg L^-1^).

**IDENTIFICATION AND QUANTIFICATION OF DESMETHYL MC-LR AND DESMETHYL MC-RR CONGENERS**

[D-Asp^3^]MC-LR standard was used for the identification and quantification of demethylated MC-LR isomers in position (3) and were called dm^3^MC-LR. The standard [D-Asp^3^]MC-RR was used for demethylated MC-RR variants in positions (3) and (5) and were called dmMC-RR (further information is included in the end of the supplementary information).

The explanation is given below:

**Desmethyl MC-LR**

Figure S9. MC-LR with aminoacids in a numbered order.

As we can see in the Figure S9 of MC-LR and as stated in bibliography, the main demethylation sites of MC-LR are:

in position (3): DMeAsp, which is named ([D-Asp^3^]MC-LR),

in position (7): Mdha (*N*-Methyldehydroalanine), which is named [Dha^7^]MC-LR

in position (5): Adda, which is named [DMAdda^5^]-MC-LR.

In the present study, for the detection of desmethyl MC-LR ([D-Asp^3^]MC-LR), the transitions from the precursor ion *m/z* 981.6 to the product ions *m/z* 135.2, 213.1 and 375.2 were monitored [1].

Product ion m/z 135 has been shown to be characteristic of PhCH_2_CH(OMe) (Adda5) fragment) for all MCs [2]. This means that the detected desmethyl MC-LR could not be the congener [DMAdda^5^]-MC-LR.

The product ions *m/z* 213.1 and 375.2 are well known ions of MCs and they are attributed to [Glu-Mdha+H]^+^ [^33^](#_ENREF_33) and [C_11_H_15_O-Glu-Mdha]^+^ [^34^](#_ENREF_34), respectively. The presence of these two product ions, is a clear indication that the Mdha(7) aminoacid is present in the methylated form. So when we are detecting these two product ion transitions for desmethyl MC-LR, we are certain that the demethylated part is not position 7 (Mdha residue). In other words, [Dha^7^]MC-LR is not the one detected based on the transitions used in LC-MS/MS.

Apart for [Dha^7^]MC-LR, three more desmethyl MC-LR congeners are currently known: (a) [D-Asp^3^]MC-LR, (b) [D-Asp^3^, (E)-Dhb^7^]MC-LR and (c) [D-Asp^3^, (Z)-Dhb^7^]MC-LR [4, 5]. Since Dhb (dehydrobutyrine) is isobaric with MDha, all these three congeners are expected to have the same fragments (based on the prediction of CyanoTox program by Dr. W.M.A. Niessen ©2001, hyphen MassSpec, the Netherlands). The congeners present the same *m/z* values for their product and precursor ions and would probably co-elute at nearly the same retention time. In order to clearly and indisputably distinguish the different desmethyl congeners which are possibly present in the samples, LC-MS/MS instrumentation would not provide enough data but the use of an aminoacid-sequencing technique (e.g. Fast Atom Bombardment FAB-MS/MS) would be necessary.

For this study, regarding desmethyl MC-LR, we only had access to certified standards for [D-Asp3]MC-LR. To the best of our knowledge, there are no commercially available standards for the rest of the desmethyl MC-LR congeners, so as to confirm the presence of these compounds and additionally to evaluate the ions ratios of the product ions for each one of the similar congeners. Therefore, the identification and quantitation of desmethyl MC-LR, was based on the available standard, more specifically on the retention time and two product ion ratios.

**Therefore, in this study [D-Asp^3^]MC-LR standard was used for quantitation and the identification of all desmethyl MC-LR congeners in position (3), which coeluted in the retention time of the standard and were called dm^3^MC-LR.**

**Desmethyl MC-RR:**


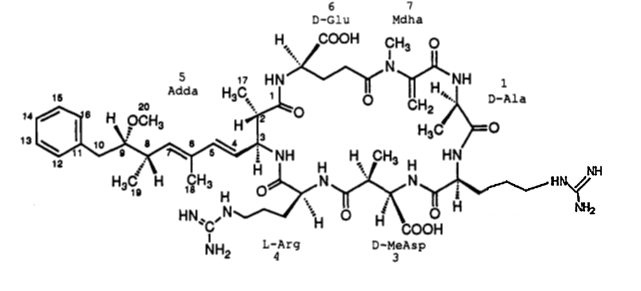


Figure S10. MC-RR with aminoacids in a numbered order

As we can see in the Figure S10 of MC-RR and as stated in bibliography, the main demethylation sites of MC-RR are:

in position (3): DMeAsp and it is called [D-Asp^3^]MC-RR,

in position (7): Mdha (*N*-Methyldehydroalanine) and it is called [Dha^7^]MC-RR,

in position (5): Adda has never been identified up to now [6].

Moreover there is an isobaric desmethyl MC-RR congener (similar to desmethyl MC-LR) which includes Dhb in position (7) and is called [D-Asp^3^, (E)-Dhb^7^]MC-RR.

In our study regarding desmethyl MC-RR, the detection was performed by monitoring the transitions from the precursor ion [M+2H]^+^ *m/z* 512.9 to the product ions *m/z* 103, 127 and 135 [^35^](#_ENREF_35).

As already mentioned, the product ion *m/z* 135 is characteristic fragment of Adda(5) moiety, attributed as [PhCH_2_CH(OMe)]^+^ [^33^](#_ENREF_33). The structures of the product ions m/z 103 and m/z 127 are not known by past bibliography, although they have been used for identification and quantification. These ions were selected in this study because, they were among the three most abundant product ions in fragmentation spectrum of [D-Asp^3^]MC-RR (Fig S11).

Figure S11: Fragmentation spectrum of [D-Asp3]MC-RR, obtained by a Finnigan TSQ Quantum Discovery Max triple-stage quadrupole mass spectrometer (Thermo, USA), equipped with electrospray ionization (ESI) source in positive mode.

According to the expected structures of the ion fragments produced in MS/MS, provided by Thermo Massfrontier ™ software, *m/z* 103 corresponds to the structure:


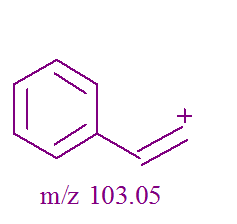
 from the Adda moiety

and m/z 127 corresponds to the structure:


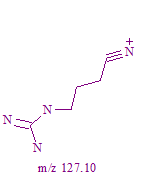
from the Arginine of position 2 or 4.

These ions are common for all the known desmethyl MC-RR congeners, they are in positions (2) and (4) which are not related to a methyl moiety, therefore they are not helpful for differentiation of the compounds.

Based on the monitored transitions and in absence of standards, the three nowadays known desmethyl MC-RR congeners (namely [D-Asp^3^]MC-RR, [Dha^7^]MC-RR and [D-Asp^3^, (E)-Dhb^7^]MC-RR) cannot be clearly distinguished.

**Therefore, in this study [D-Asp^3^]MC-RR standard was used for quantitation and the identification of all desmethyl MC-LR congeners in positions (3) and (5), which coeluted in the retention time of the standard and were called dmMC-RR.**

**REFERENCES in supplementary material**

1 Zervou, S.-K., Christophoridis, C., Kaloudis, T., Triantis, T. M. & Hiskia, A. New SPE-LC-MS/MS method for simultaneous determination of multi-class cyanobacterial and algal toxins. *J. Hazard. Mater.* **323, Part A**, 56-66, doi:<http://dx.doi.org/10.1016/j.jhazmat.2016.07.020> (2017).

2 Moustaka-Gouni, M. *et al.* First report of Aphanizomenon favaloroi occurrence in Europe associated with saxitoxins and a massive fish kill in Lake Vistonis, Greece. *Mar.Freshwater Res.* **68**, 793-800, doi:10.1071/MF16029 (2017).

3 Gkelis, S., Lanaras, T., Sivonen, K. & Taglialatela-Scafati, O. Cyanobacterial toxic and bioactive peptides in freshwater bodies of Greece: Concentrations, occurrence patterns, and implications for human health. *Mar. Drugs* **13**, 6319-6335, doi:10.3390/md13106319 (2015).

4 Gkelis, S. & Zaoutsos, N. Cyanotoxin occurrence and potentially toxin producing cyanobacteria in freshwaters of Greece: A multi-disciplinary approach. *Toxicon* **78**, 1-9, doi:<http://dx.doi.org/10.1016/j.toxicon.2013.11.010> (2014).

5 Gkelis, S., Papadimitriou, T., Zaoutsos, N. & Leonardos, I. Anthropogenic and climate-induced change favors toxic cyanobacteria blooms: Evidence from monitoring a highly eutrophic, urban Mediterranean lake. *Harmful Algae* **39**, 322-333, doi:<http://dx.doi.org/10.1016/j.hal.2014.09.002> (2014).

6 Berillis, P., Papadimitriou, T., Petridou, E., Kormas, K. & Kagalou, I. Brain and liver histopathological examination of carassius gibelio from a newly reconstructed lake with toxic cyanobacteria. *Turkish Journal of Fisheries and Aquatic Sciences* **14**, 213-219 (2014).

7 Kaloudis, T. *et al.* Determination of microcystins and nodularin (cyanobacterial toxins) in water by LC–MS/MS. Monitoring of Lake Marathonas, a water reservoir of Athens, Greece. *J. Hazard. Mater.* **263, Part 1**, 105-115, doi:<http://dx.doi.org/10.1016/j.jhazmat.2013.07.036> (2013).

8 Papadimitriou, T., Katsiapi, M., Kormas, K. A., Moustaka-Gouni, M. & Kagalou, I. Artificially-born “killer” lake: Phytoplankton based water quality and microcystin affected fish in a reconstructed lake. *Science of The Total Environment* **452–453**, 116-124, doi:<http://dx.doi.org/10.1016/j.scitotenv.2013.02.035> (2013).

9 Oikonomou, A., Katsiapi, M., Karayanni, H., Moustaka-Gouni, M. & Kormas, K. A. Plankton microorganisms coinciding with two consecutive mass fish kills in a newly reconstructed lake. *The Scientific World Journal* **2012** (2012).

10 Lymperopoulou, D. S., Kormas, K. A. & Karagouni, A. D. Variability of Prokaryotic Community Structure in a Drinking Water Reservoir (Marathonas, Greece). *Microbes and Environments* **27**, 1-8, doi:10.1264/jsme2.ME11253 (2012).

11 Papadimitriou, T., Armeni, E., Stalikas, C. D., Kagalou, I. & Leonardos, I. D. Detection of microcystins in Pamvotis lake water and assessment of cyanobacterial bloom toxicity. *Environ. Monit. Assess.* **184**, 3043-3052, doi:10.1007/s10661-011-2169-5 (2012).

12 Lymperopoulou, D., Kormas, K., Moustaka-Gouni, M. & Karagouni, A. Diversity of cyanobacterial phylotypes in a Mediterranean drinking water reservoir (Marathonas, Greece). *Environmental Monitoring and Assessment* **173**, 155-165, doi:10.1007/s10661-010-1378-7 (2011).

13 Dimitrakopoulos, I. K., Kaloudis, T. S., Hiskia, A. E., Thomaidis, N. S. & Koupparis, M. A. Development of a fast and selective method for the sensitive determination of anatoxin-a in lake waters using liquid chromatography-tandem mass spectrometry and phenylalanine-d 5 as internal standard. *Anal. Bioanal. Chem.* **397**, 2245-2252, doi:10.1007/s00216-010-3727-3 (2010).

14 Papadimitriou, T., Kagalou, I., Bacopoulos, V. & Leonardos, I. D. Accumulation of microcystins in water and fish tissues: An estimation of risks associated with microcystins in most of the Greek Lakes. *Environ. Toxicol.* **25**, 418-427 (2010).

15 Triantis, T. *et al.* Development of an integrated laboratory system for the monitoring of cyanotoxins in surface and drinking waters. *Toxicon* **55**, 979-989, doi:10.1016/j.toxicon.2009.07.012 (2010).

16 Michaloudi, E., Moustaka-Gouni, M., Gkelis, S. & Pantelidakis, K. Plankton community structure during an ecosystem disruptive algal bloom of Prymnesium parvum. *Journal of Plankton Research* **31**, 301-309 (2009).

17 Vareli, K., Pilidis, G., Mavrogiorgou, M.-C., Briasoulis, E. & Sainis, I. Molecular characterization of cyanobacterial diversity and yearly fluctuations of Microcystin loads in a suburban Mediterranean Lake (Lake Pamvotis, Greece). *J. Environ. Monit.* **11**, 1506-1512, doi:10.1039/b903093j (2009).

18 Kagalou, I., Papadimitriou, T., Bacopoulos, V. & Leonardos, I. Assessment of microcystins in lake water and the omnivorous fish (Carassius gibelio, Bloch) in Lake Pamvotis (Greece) containing dense cyanobacterial bloom. *Environ. Monit. Assess.* **137**, 185-195, doi:10.1007/s10661-007-9739-6 (2008).

19 Moustaka-Gouni, M., Vardaka, E. & Tryfon, E. Phytoplankton species succession in a shallow Mediterranean lake (L. Kastoria, Greece): Steady-state dominance of Limnothrix redekei, Microcystis aeruginosa and Cylindrospermopsis raciborskii. *Hydrobiologia* **575**, 129-140 (2007).

20 Moustaka-Gouni, M. *et al.* Plankton food web structure in a eutrophic polymictic lake with a history of toxic cyanobacterial blooms. *Limnol. Oceanogr.* **51**, 715-727 (2006).

21 Gkelis, S., Lanaras, T. & Sivonen, K. The presence of microcystins and other cyanobacterial bioactive peptides in aquatic fauna collected from Greek freshwaters. *Aquat. Toxicol.* **78**, 32-41, doi:<http://dx.doi.org/10.1016/j.aquatox.2006.02.001> (2006).

22 Gkelis, S., Moustaka-Gouni, M., Sivonen, K. & Lanaras, T. First report of the cyanobacterium Aphanizomenon ovalisporum Forti in two Greek lakes and cyanotoxin occurrence. *Journal of Plankton Research* **27**, 1295-1300, doi:10.1093/plankt/fbi085 (2005).

23 Gkelis, S. *et al.* Limnothrix redekei (Van Goor) Meffert (Cyanobacteria) Strains from Lake Kastoria, Greece Form a Separate Phylogenetic Group. *Microb Ecol* **49**, 176-182, doi:10.1007/s00248-003-2030-7 (2005).

24 Gkelis, S., Harjunpää, V., Lanaras, T. & Sivonen, K. Diversity of hepatotoxic microcystins and bioactive anabaenopeptins in cyanobacterial blooms from Greek freshwaters. *Environ. Toxicol.* **20**, 249-256 (2005).

25 Cook, C. M., Vardaka, E. & Lanaras, T. Toxic Cyanobacteria in Greek Freshwaters, 1987—2000: Occurrence, Toxicity, and Impacts in the Mediterranean Region. *Acta Hydroch. Hydrob.* **32**, 107-124, doi:10.1002/aheh.200300523 (2004).

26 Gkelis, S., Vardaka, E., Moustaka-Gouni, M., Lanaras, T. in *7th International Phycological Congress, Thessaloniki, Greece.* Vol. 25. Phycologia 40, 123 (ed M. Moustaka-Gouni, Bird, C. J., Cox, E. J., Raven, J. A., Lanaras, T., Karpouchtsis, J., Simpson, G. E., Mann, D. G.) (2001).

27 Gkelis, S., Harjunpää, V., Vardaka, E., Lanaras, T., Sivonen, K. . in *5th International Conference on Toxic Cyanobacteria* (Noosa, Queensland, Australia, 2001).

28 Lanaras, T. & Cook, C. M. Toxin extraction from an Anabaenopsis milleri — dominated bloom. *Sci. Total Environ.* **142**, 163-169, doi:<http://dx.doi.org/10.1016/0048-9697(94)90324-7> (1994).

29 Lanaras, T., Tsitsamis, S., Chlichlia, C. & Cook, C. M. Toxic cyanobacteria in Greek freshwaters. *J. Appl. Phycol.* **1**, 67-73 (1989).

30 WHO. Guidelines for Safe Recreational Water Environments. Volume 1. Coastal and Fresh Waters. (Geneva, Switzerland, 2003).

31 Farrer, D., Counter, M., Hillwig, R. & Cude, C. Health-based cyanotoxin guideline values allow for cyanotoxin-based monitoring and efficient public health response to cyanobacterial blooms. *Toxins (Basel)* **7**, 457-477, doi:10.3390/toxins7020457 (2015).

32 WHO. Guidelines for drinking-water quality, 4th edition, incorporating the 1st addendum. (Geneva, Switzerland, 2011).

33 Namikoshi, M. *et al.* Identification of 12 hepatotoxins from a homer lake bloom of the cyanobacteria Microcystis aeruginosa, Microcystis viridis, and Microcystis wesenbergii: Nine new microcystins. *Journal of Organic Chemistry* **57**, 866-872 (1992).

34 Edwards, C. *et al.* Analysis of microcystins from cyanobacteria by liquid chromatography with mass spectrometry using atmospheric-pressure ionization. *Rapid Communications in Mass Spectrometry* **7**, 714-721, doi:10.1002/rcm.1290070807 (1993).

35 Zervou, S.-K., Christophoridis, C., Kaloudis, T., Triantis, T. M. & Hiskia, A. New SPE-LC-MS/MS method for simultaneous determination of multi-class cyanobacterial and algal toxins. *Journal of Hazardous Materials* **323**, 56-66, doi:https://doi.org/10.1016/j.jhazmat.2016.07.020 (2017).
